# Effect of Molecular Organization on the Properties of Fractionated Lignin-Based Thiol–Ene Thermoset Materials

**DOI:** 10.1021/acsomega.3c03022

**Published:** 2023-07-03

**Authors:** Iuliana Ribca, Benedikt Sochor, Stephan V. Roth, Martin Lawoko, Michael A. R. Meier, Mats Johansson

**Affiliations:** † Wallenberg Wood Science Center (WWSC), Department of Fibre and Polymer Technology, KTH Royal Institute of Technology, Teknikringen 56-58, SE-100 44 Stockholm, Sweden; ‡ Division of Coating Technology, Department of Fibre and Polymer Technology, KTH Royal Institute of Technology, Teknikringen 48, SE-100 44 Stockholm, Sweden; § Deutsches-Elektronen Synchrotron (DESY), 22607 Hamburg, Germany; ∥ Division of Wood Chemistry and Pulp Technology, Department of Fibre and Polymer Technology, KTH Royal Institute of Technology, Teknikringen 56, SE-100 44 Stockholm, Sweden; ⊥ Institute of Organic Chemistry (IOC), Materialwissenschaftliches Zentrum MZE, Karlsruhe Institute of Technology (KIT), Straße am Forum 7, 76131 Karlsruhe, Germany; # Institute of Biological and Chemical Systems-Functional Molecular Systems (IBCS-FMS), Karlsruhe Institute of Technology (KIT), Hermann-von-Helmholtz-Platz 1, 76344 Eggenstein-Leopoldshafen, Germany;

## Abstract

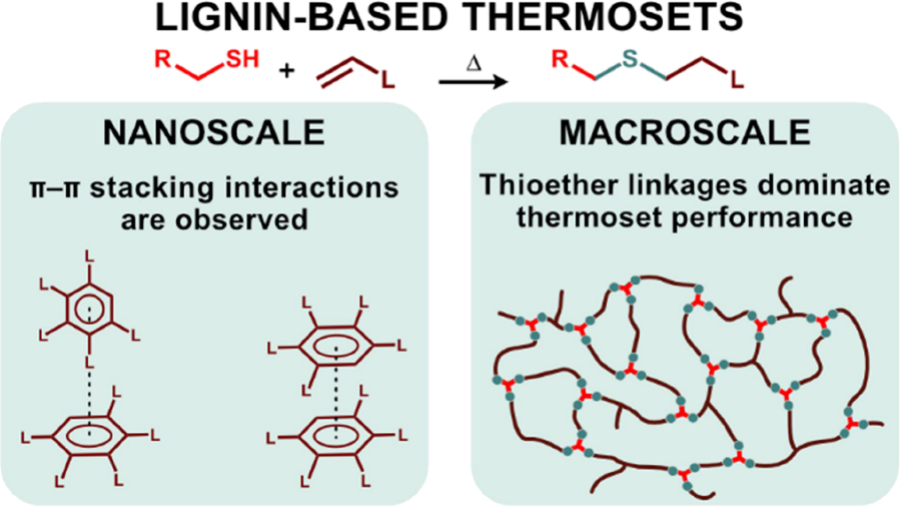

In this study, the combination of sequential solvent fractionation of technical Kraft lignin was followed by allylation of most OH functionalities to give highly functional thermoset resins. All lignin fractions were highly functionalized on the phenolic (≥95%) and carboxylic acid OH (≥85%) and to a significant extent on the aliphatic OH moieties (between 43 and 75%). The resins were subsequently cross-linked using thiol–ene chemistry. The high amount of allyl functionalities resulted in a high cross-link density. Dynamic mechanical analysis measurements showed that the thioether content, directly related to the allyl content, strongly affects the performance of these thermosets with a glass transition temperature (*T*_g_) between 81 and 95 °C and with a storage modulus between 1.9 and 3.8 GPa for all thermosets. The lignin fractions and lignin-based thermosets’ morphology, at the nanoscale, was studied by wide-angle X-ray scattering measurements. Two π–π stacking interactions were observed: sandwich (≈4.1–4.7 Å) and T-shaped (≈5.5–7.2 Å). The introduction of allyl functionalities weakens the T-shaped π–π stacking interactions. A new signal corresponding to a distance of ≈3.5 Å was observed in lignin-based thermosets, which was attributed to a thioether organized structure. At the same time, a lignin superstructure was observed with a distance/size corresponding to 7.9–17.5 Å in all samples.

## Introduction

The continuous need to develop new bio-based materials is driven by the decreasing amount of fossil reserves as well as environmental concerns. In particular, in the field of polymeric materials, which is dominated by petroleum-based raw chemicals, biomass-derived alternatives are urgently needed for the shift toward a more sustainable global carbon economy.^1−3^ A very promising alternative to resources gathered from crude oil is technical lignin, a byproduct of pulp and paper industries. Lignin is the most abundant natural aromatic resource, with an estimated annual production of 70 million tons of technical lignin from pulp and paper facilities worldwide.^4^ Today, however, technical lignin is mainly used as a source of energy by the pulp and paper industry,^3^ hence underutilizing the potential value of this resource.

Lignin is a complex polyphenol biopolymer composed mainly of three phenolic structures, i.e., guaiacyl (G), syringyl (S), and *p*-hydroxyphenyl (H) units with varying ratios depending on the lignin source.^5^ Most common inter-unit linkages present in native softwood lignin are β-O-4, β-5, and 5-5.^6−9^ However, these are severely degraded in the Kraft process, and only a few percent are left in Kraft lignin. The aryl ethers are hydrolyzed to form new phenolic ends in lignin while the β-5 will react to form stilbene structures through the loss of methanol from the gamma carbon. Retro-aldol reactions are also reported, yielding the loss of side aliphatic groups and leaving aromatic end groups. These aromatic end groups condense through radical couplings to form new carbon–carbon bonds between aromatic units, e.g., 5-5′ and 1-5′.^10,11^ Overall, the structural features of Kraft lignin are significantly different from those of native lignins and remain a challenge to fully assign by existing analytical methods. Lignin also contains a variety of functional groups. The main chemical functional groups are hydroxyl (phenolic, aliphatic, and carboxylic), methoxy, carbonyl, and carboxyl groups.^8,12^

The chemical structure of technical lignin strongly depends on two factors: the retrieval process and the plant source. At present, four methods are used industrially for the isolation of lignin: the Kraft, sulfite (sulfur-based), soda, and organosolv (sulfur-free) processes, with the Kraft process being the dominant procedure.^4^ These methods all influence the obtained chemical structure of technical lignin to a different extent. This leads to heterogeneous structures, varying in terms of molecular weight, dispersity, and functional groups for every batch.^3^ The structural differences have a strong impact on the solubility, thermal stability, mechanical properties, and inter- and/or intra-molecular interactions (hydrogen bonds and π–π stacking interactions) of technical lignin and consequently on its reactivity.^13,14^ This results in major challenges if technical lignin resources are subjected to a subsequent chemical modification via a standardized process. Chemical modifications, however, are often necessary to make the lignin more compatible and reactive, allowing it to be incorporated into polymeric systems.^3,15^ One way to tackle this challenge is to fractionate lignin to render it more homogeneous and consequently more reproducible properties.^16,17^ Several approaches have been developed, such as solvent extraction (partial solubility in organic solvents),^18−21^ solvent/water extraction (partial solubility in different ratios of organic/water solution),^22,23^ pH control method (changing the pH of alkali-soluble lignin),^24^ membrane filtration (physical filtration),^25,26^ or microwave processing (partial solubility in different solvents).^27^ Another approach is depolymerization or fragmentation of lignin to form monomeric units, such as phenol or vanillin, which can be used as conventional building blocks.^28−30^ Several pathways have been demonstrated for chemical modification of lignin: introducing new chemically active sites (amination,^31^ sulfonation,^32^ etc.), modifying the hydroxyl groups (allylation,^33−35^ esterification,^36^ phenolation,^37^ etc.), and synthesizing graft copolymers.^29,38−41^ Recently, it was shown that lignin can be first functionalized by using a scalable one-pot method and consequently fractionated through downward precipitation.^42^

The incorporation of allyl groups onto technical lignin represents a promising pathway to create reactive lignin derivatives, suitable for radical polymerization.^43^ The traditional way of introducing allyl groups on lignin is an etherification reaction using halogenated allyl compounds such as allyl bromide^33^ or allyl chloride.^35,44^ These allylated products represent promising materials for further utilization of technical lignin as a thiol–ene thermoset component.^35,45,46^ These thermosets show a higher glass transition temperature compared with aliphatic systems due to their relatively high aromaticity.^47,48^ It was also shown that the final properties of the lignin-based thiol–ene thermosets can be tuned by varying the partially allylated lignin fractions or thiol cross-linker.^45,46^ In 2016, Over and Meier presented a more sustainable allylation method for organosolv lignin using diallyl carbonate (DAC), where almost all the hydroxyl groups could be allylated.^34^ These allyl-functional resins were also evaluated as thermoset resins in combination with vegetable oils using metathesis chemistry.^49^ Additional reported methods to introduce vinyl groups onto lignin involve using acryloyl chloride,^50^ methacryloyl chloride,^51^ allyl alcohol,^52^ vinyl ethylene carbonate,^53^ or a two-step route using ethylene carbonate and acrylic acid.^43^

In the present study, softwood Kraft lignin (LignoBoost) is utilized to prepare thiol–ene thermosetting materials with a higher allyl functionality compared to previously studied thermosets based on the same technical lignin. First, washed technical softwood lignin was sequentially solvent fractionated, then allylated using DAC, and finally mixed with a trifunctional thiol cross-linker to be thermally cured (Scheme S1). Furthermore, an extensive study using wide-angle X-ray scattering (WAXS) was performed, both on the resins and final thermosets, revealing well-ordered molecular structures of the lignin.

Previous studies have revealed details on the effect of different fractions and different cross-linker structures on the final thermoset performances when partially modifying the lignin with allyl-aryl ethers.^45,46^ The main objective of the present study is to obtain a more holistic understanding of how different structural features in relation to each other affect the performance of lignin-based thermosets. Parameters such as relative amounts of functional groups (aliphatic OH, different phenolic OH, carboxylic acid OH, allyl-ether, and allyl-ester), molecular weight, cross-link density, and thioether content and the effect of these variables on the morphology and mechanical properties were investigated. The results provide an insight on both how an increased functionality affects the final structures and how the removal of polar OH groups within the thermoset structure influences their properties.

## Experimental Section

### Materials

Softwood (SW) LignoBoost Kraft lignin as a fine powder was kindly donated by Stora Enso (Finland). Ethyl acetate (EtOAc, ≥99%), ethanol (EtOH, ≥99.8%), methanol (MeOH, ≥99.8%), acetone (≥99.5%), and hydrochloric acid (HCl, 37%) were purchased from VWR International. Lithium hydroxide (LiOH, >98%), DAC (99%), tetrabutylammonium bromide (TBAB, 98%), cyclohexane (99.5%), and trimethylolpropane tris(3-mercaptopropionate) (3TMP, ≥95%) were purchased from Sigma-Aldrich. Magnesium sulfite (MgSO_4_, 99%) was purchased from Thermo Scientific Chemicals. Silastic T-2 base/curing agent (10:1 w/w) was used to prepare the silicone molds. It was obtained from Dow Corning. All other chemicals were purchased from Sigma-Aldrich and used as received.

### Procedures

#### Initial Purification of Technical Lignin Powder

Softwood Kraft lignin powder was washed prior to the fractionation. The procedure is described in the SI (page 5).^46^ The washed lignin was denoted as SW-Initial.

#### Lignin Extraction Using the Sequential Solvent Fractionation Approach

Solvent fractionation of the technical lignin was performed according to a previously reported method and is briefly described in the SI (page 5).^19,46^ The obtained fractions were named SW-EtOAc, SW-EtOH, SW-MeOH, SW-Acetone, and SW-Insoluble. The last fraction, SW-Insoluble, was not soluble in the four previously mentioned solvents; however, it is soluble in dimethyl sulfoxide (DMSO), which enables its characterization.

#### Lignin Allylation

Allylated lignins were prepared from all five obtained lignin fractions as well as the washed initial lignin based on a previously reported protocol. The procedure is described in the SI (page 5).^34^ The samples were denoted as DAC-SW-Initial, DAC-SW-Solvent (solvent = EtOAc, EtOH, MeOH, and Acetone), and DAC-SW-Insoluble. The allyl content was determined by calculating the difference between the total OH content before and after allylation. It was reported as the mmol of various allyl functionalities/g lignin.

#### Decarboxylation of Allylated Lignin Fractions

Decarboxylation was performed following a general procedure exemplified in the SI (page 6).^34^ The obtained samples were denoted as D-DAC-SW-Initial, D-DAC-SW-Solvent (solvent = EtOAc, EtOH, MeOH, and Acetone), and D-DAC-SW-Insoluble.

#### Preparation of Lignin-Based Thermosets

Six different thermoset samples were prepared following the procedure described earlier by Jawerth et al. using 3TMP as a cross-linking agent, which is presented in the SI (page 6).^35^ The equivalent ratio between the reactive groups (ene/thiol) was kept at 1:1. All resin formulations and thermosets were denoted in the following way: T3-DAC-SW-Initial, T3-DAC-SW-Solvent (solvent = EtOAc, EtOH, MeOH, and Acetone), and T3-DAC-SW-Insoluble. Full details of the sample compositions can be found in Table S1.

#### Characterization Techniques

All the characterization techniques used in this study are presented in the SI.

## Results and Discussion

The heterogeneity of technical lignins, obtained from different processes, makes it difficult to predict the final properties of thermoset materials derived from these sources. It is, furthermore, difficult to interpret structure–property relationships in these systems, since many parameters normally vary at the same time. The present work aims to elucidate these structure–property relationships by studying a series of novel allylated lignins and comparing them with previously described thermosets.

### Technical Lignin Fractionation

In the first step, Kraft lignin was washed with water to lower its ash content. The inorganic content before and after washing was reported in a previous study.^46^ Technical lignin is a heterogeneous and complex mixture with a broad distribution of molar masses. A sequential solvent fractionation approach was used in this study, allowing the retrieval of more uniform lignin fractions with specific characteristics. The different lignin fractions were analyzed in terms of predominant linkages,^11,54^ OH content, thermal behavior, and molecular weight distribution.^45,46,55,56^

**Figure 1:**
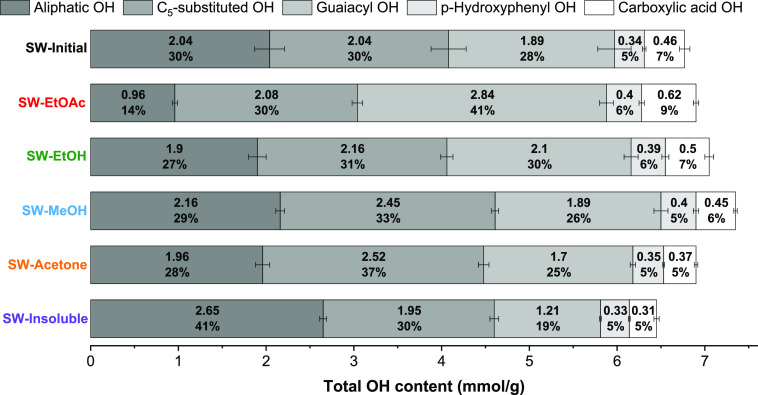
Quantification of various OH functionalities of fractionated SW-Lignin determined by ^31^P NMR. Lignin samples were derivatized with 2-chloro-4,4,5,5-tetramethyl-1,3-2-dioxaphospholane (TMDP) using *N*-hydroxy-5-norbornene-2,3-dicarboximide (NHND) as the internal standard. Data were presented as relative percentages for each fraction as well as absolute values in mmol of the different OH groups/g lignin.

The yield of SW-EtOAc was 23 ± 1%, for SW-EtOH, it was 22 ± 1%, for SW-MeOH, it was 8 ± 1%, and for SW-Acetone, it was 10 ± 1% (Table S2). The *M*_w_ for all recovered lignin fractions increases from SW-EtOAc (1250 ± 50 g/mol) to SW-Insoluble (27,050 ± 5150 g/mol). *Đ* was found to decrease for all fractions to 1.7 ± 0.2 (apart from SW-Insoluble, for which it was 3.4 ± 0.2) compared to the 3.2 ± 0.1 of the starting lignin (Figure S1, Table S2). However, the data in Table S2 provide relative values because of a lack of calibration standards with high structural similarity. The OH content of the different lignin fractions was determined by ^31^P NMR spectroscopy, and this is shown in Figure 1. Various types of OH groups that can be distinguished and quantified using this method are aliphatic, C_5_-substituted, guaiacyl, *p*-hydroxyphenyl, and carboxylic acid OH.^57^ It was found that the total phenolic OH content (especially guaiacyl OH) and the carboxylic acid OH content were higher in the SW-EtOAc fraction, while the aliphatic OH content was higher in SW-Insoluble.^11,46,55,56^ A high functionalization of all OH groups can thus provide information on the structure–property relationship when comparing with previously published data.^45,46^

The lignin fractionation approach allows different fractions with specific structures to be evaluated. It also represents a promising route for retrieving a broad variety of lignins, which can be used for resin preparation.

### Allylation of Softwood Lignin

**Scheme 1:**
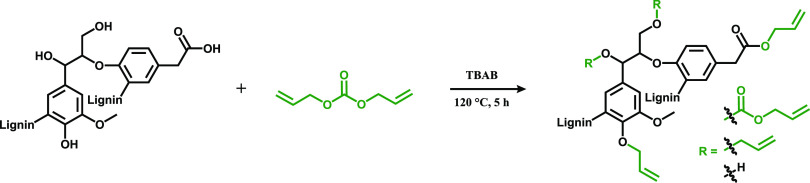
Allylation of SW-Lignin Samples with DAC in the Presence of TBAB

The initial lignin and the retrieved fractions were further used for chemical modification with DAC. DAC was used as an allylation reagent in order to minimize the use of halogenated reagents and reduce the use of hazardous substances. All in-going reagents are considered to be on an irritant level at most and not directly toxic, as shown by data found in the MSDS.^58−60^ The allylation reaction with DAC takes place at a relatively low temperature (120 °C); it is relatively fast (5 h) and does not lead to detectable side reactions such as cross-linking between vinyl groups. This reaction does not require any solvent where TBAB acts as a phase transfer catalyst during the reaction, and it can be recovered and reused.^34^ DAC can be synthesized from dimethyl carbonate, which is renewable, biodegradable, less toxic, and can be synthesized using CO_2_ as the building block.^34^ However, the reaction releases some amount of allyl alcohol, a toxic compound.^53,61^ The allylation reaction was studied by size exclusion chromatography (SEC), ^1^H NMR, ^31^P NMR, and Fourier transform infrared spectroscopy (FTIR). A general representation of the allylation reaction of SW-Lignin is shown in Scheme 1.

Figure S2 and Table S3 show the SEC results of the allylated lignin samples, and the detailed values for *M*_n_, *M*_w_, and *Đ* are listed. The *M*_w_ of allylated lignin samples increased after modification as a result of incorporation of the allyl functionalities. At the same time, an anomalous behavior was observed in the trend, where *M*_n_ values decrease for most samples. The reason for this behavior is unclear, but it is proposed to be due to a combination of factors, e.g., polarity, solvent-resin interactions, and resin-column material interactions. The SEC data should thus be considered as trends, rather than absolute values. The DAC-SW-Insoluble fraction did not fully dissolve in DMSO; thus, the SEC data for this are omitted.

^1^H NMR spectra of unmodified and allylated lignin fractions are shown in Figure S3. The proton signals resulting from the allyl group (δ = 6.28–5.53, 5.53–4.76, and 4.76–4.07 ppm, Table S4) were found in all samples, confirming that all lignin fractions were successfully chemically modified. The signal at δ = 8.00–6.28 ppm was assigned to aromatic protons and the signal at δ = 4.07–3.47 ppm to the protons in the methoxy group, respectively.^33,34,46^

**Figure 2:**
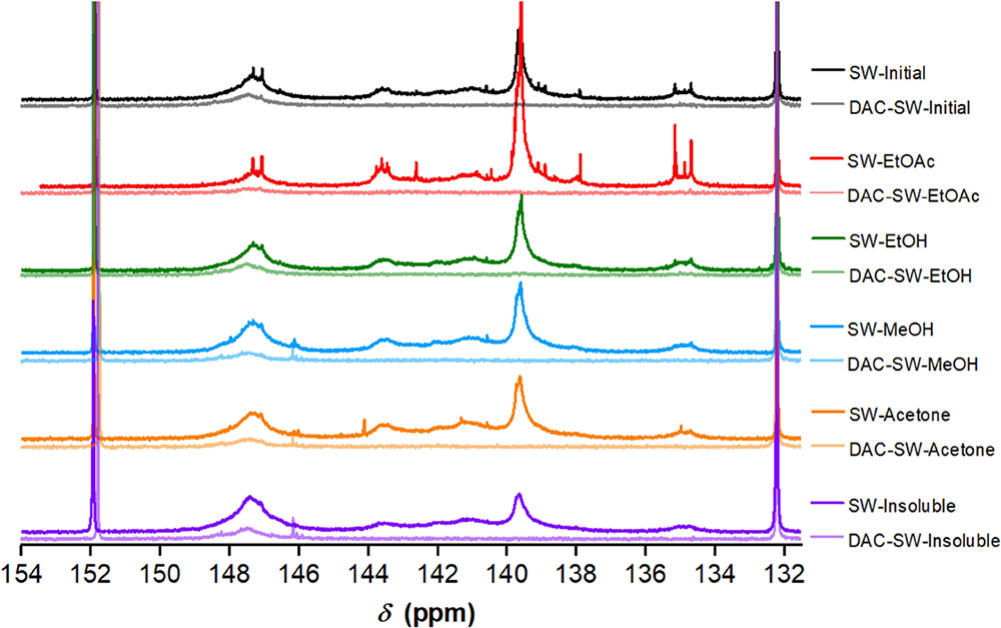
^31^P NMR spectra comparison between SW-lignin samples before and after the modification with DAC. Lignin samples were derivatized with 2-chloro-4,4,5,5-tetramethyl-1,3-2-dioxaphospholane (TMDP) using *N*-hydroxy-5-norbornene-2,3-dicarboximide (NHND) as the internal standard. The signal at around 132.2 ppm corresponds to the phosphitylation product of TMDP with water, and the signal at 151.9 ppm corresponds to the product of TMDP and NHND.

For all lignin samples, a high conversion of the total phenolic OH, ≥95% (δ = 144.7–137.0 ppm), and of carboxylic acid OH, ≥85% (δ = 136.0–133.6 ppm), was observed by ^31^P NMR (the spectra are shown in Figure 2). The signal of the aliphatic OH was drastically reduced in the ^31^P NMR spectra of allylated lignin (δ = 149.5–145.5 ppm). Consequently, the data showed that aliphatic OH groups were only partially modified (with a conversion between 43 and 75%) and that ≥80% of total OH groups were allylated (Table S5).

The FTIR spectra of lignin fractions before and after allylation showed structural differences between them, as can be seen in Figure S5 for the EtOH fraction and in Figure S6 for all other fractions. The broad signal between 3700 and 3100 cm^–1^ was assigned to hydrogen bonds of phenolic groups, OH stretching in phenols, alcohols, acids, and present water.^62^ FTIR measurements revealed a strong decrease in absorbance of this signal due to the functionalization of the hydroxyl groups. The new signals at 3078, 1647, 986, and 923 cm^–1^ were attributed to the stretching and deformation vibration of the allyl functionalities.^33,34^ In addition, these spectra also showed a new signal at 1743 cm^–1^, which was attributed to the carbonyl C=O bond stretching vibration signal resulting from allyl carbonates installed at aliphatic OH groups.^63^ The absorption signals at 1595 (before allylation), 1581 (after allylation), and 1509 cm^–1^ corresponding to the aromatic ring stretching vibrations were present in the spectra.^64^ The signal present at 1793 cm^–1^ corresponds to small amounts of cyclic carbonates formed due to side reactions.^65^

It was found that more than 85% of TBAB could be recovered without impurities as shown by ^1^H NMR spectroscopy, comparing the recycled TBAB to the initial one (Figure S9).

The thermal behavior of the allylated lignin fractions was investigated by differential scanning calorimetry (DSC) (Figure S10) and thermogravimetric analysis (TGA) (Figure S11). All allylated lignin fractions exhibited single and broad glass transition temperatures, which are significantly lower than the *T*_g_ of the unmodified lignin.^46^ The *T*_g_ is influenced by the molecular weight, hydrogen bonds, and chemical structure.^66^ By modifying the OH groups, the hydrogen bonding capacity was lowered, causing a reduction in *T*_g_. At the same time, by introducing different allyl functionalities, the free volume as well as the molecular mobility increases. As a result, *T*_g_ of the allylated lignin is lower than 100 °C for all fractions. All allylated lignin samples showed thermal stability up to 150 °C. It has previously been shown that a Claisen rearrangement starts around 150 °C, but this should not result in weight loss.^33,44,67^ The first weight loss occurs in the range of 150–300 °C and can be attributed to the propanoid side chain cleavage.^68^ The second weight loss takes place between 300 and 600 °C due to rearrangements of the aromatic rings.^69^

### Decarboxylation of Allylated Lignin

In a previous study on organosolv lignin, it was shown that the phenolic OH groups were converted to the corresponding allyl ethers and that aliphatic OH groups were partially etherified and partially carboxyallylated.^34^ Another study on ferulic acid showed that the carboxylic acid OH groups were converted to the corresponding allyl esters.^67^ In order to determine whether hydroxyl groups were etherified or carboxyallylated, allylated lignin samples were treated with LiOH. Under this basic condition, the potentially formed carbonates and esters will be cleaved (Scheme S2).^34^^31^P NMR analysis showed that after the treatment with LiOH, the content of the allylated aliphatic OH decreased between 5 and 35%; meanwhile, carboxylic OH groups were fully regenerated (Table S6 and Figure S12). The amount of allylated phenolic OH did not change after the treatment with LiOH. Moreover, ^1^H NMR results showed a decrease of the allylic proton integrals after the treatment with LiOH to the unchanged aromatic protons (Figure S13). FTIR studies were also done in order to investigate the decarboxylation reaction (Figure S14). A comparison between allylated lignin samples before and after the treatment with LiOH indicated that the OH signal increased but was still lower than that for the unmodified samples, which can be due to the partial regeneration of the aliphatic OH groups and full regeneration of carboxylic acid OH groups. The results of the decarboxylation reaction also confirmed the absence of the signal at 1734 cm^–1^. These results provide proof that, in addition to the etherification of the phenolic OH groups, aliphatic OH groups were partially carboxyallylated and partially etherified and that carboxylic OH groups were converted into allyl esters.

### Curing Performance of Lignin-Based Thermosetting Resins

**Figure 3:**
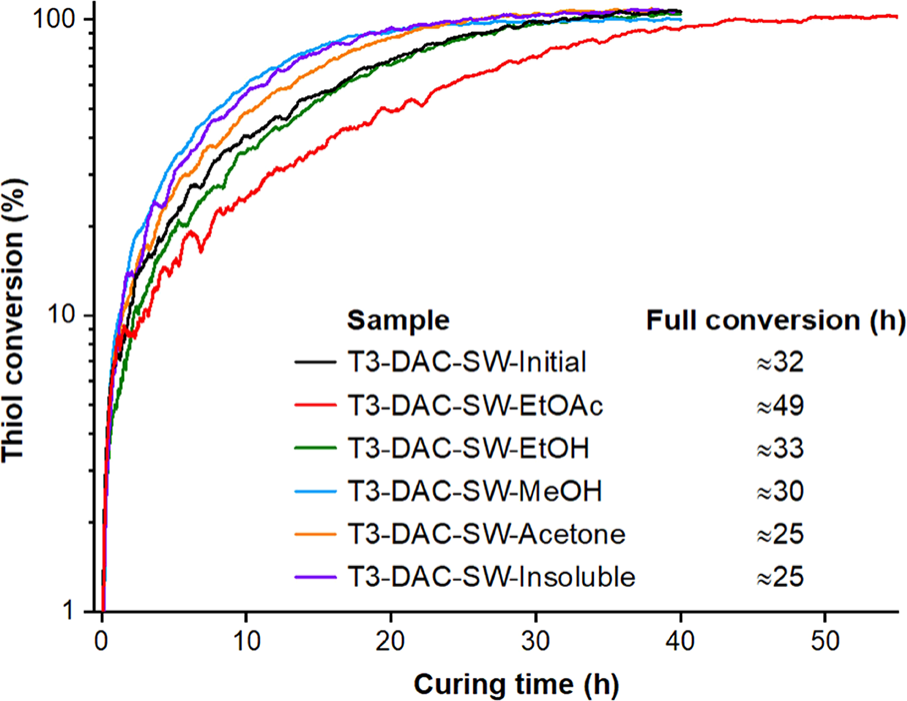
Conversion of the −SH signal at 2607–2533 cm^**–**1^ during thermal curing at 125 °C as a function of time for a 1:1 thiol/ene mixture, determined by RT-FTIR.

Thermal curing of DAC-Lignins with a trifunctional thiol cross-linker (3TMP) was followed by real-time FTIR (RT-FTIR) at 125 °C. The spectra before and after curing for all formulations are shown in Figures S15 and S16 with zooming into the regions of thiol (2571 cm^–1^) and ene (1647 cm^–1^) signals. The conversion of thiol functional groups was monitored and is reported in Figures 3 and S17. It was found that most allylated fractions exhibited a similar curing behavior, as shown in Table S7. Full conversion of thiol groups was achieved between ≈25 and 33 h, except for the DAC-SW-EtOAc formulation, which cured slower (≈50 h). The explanation for this difference is not clear, but one can notice that this fraction has a higher guaiacyl content compared to the others (higher total phenolic content by 0.5 mmol/g lignin). A high guaiacyl content has previously been proposed to lead to stronger intra/inter-molecular interactions, making functional groups less accessible/reactive.^70,71^ It also has a higher content of carboxylic acid OH and a lower amount of aliphatic OH, as shown in Figure 1.

### Morphological Characterization Using X-ray Scattering

Hydrogen bonding, π–π stacking interactions between lignin aromatic rings, lignin chain conformation (their flexibility), and degree of branching strongly influence the lignin morphology.^70^ Previous studies on lignin structures have revealed the presence of short distance ordering in the range of 4.0–4.2 and 5.3–6.1 Å considered to be related to sandwiched and T-shaped π–π stacking interactions, respectively.^72,73^ Six predominant distances/sizes (*D*_1_, *D*_2_, *D*_3_, *D*_4_, *D*_II order3_, and *D*_II order4_) were observed in lignin samples by WAXS, as shown in Table 1 and Figures S19–S24. Based on the distance within these repeating features, *D*_2_ and *D*_3_ can be attributed to T-shaped and sandwich π–π stacking (combined parallel displaced and cofacial parallel stacked^74^) interactions between the abundant lignin aromatic rings.^45,75−77^ This should however be considered a simplification due to the highly complex three-dimensional lignin network structures. These π–π stacking interactions between aromatic rings normally play an important role in the stability of the systems and their properties.^74,78,79^ It is however not clear how these internal stacking interactions scale when being imbedded into a larger structural ordering. *D*_II order 3_ and *D*_II order 4_ (*D*_II order 3 or 4_ = *D*_3 or 4_/2) were attributed to be the so-called second-order signal of *D*_3_ and *D*_4_. These signals can arise as a secondary interference pattern of the same structures and indicate a very well-ordered system.

**Table 1:** Repeating Features within SW-Lignin, DAC-SW-Lignin, and T3-DAC-SW-Lignin Samples Determined by WAXS^a^

sample	*D*_1 max_ (Å)	*D*_2 max_ (Å)	*D*_3 max_ (Å)	*D*_II order 3_ (Å)
SW-Initial	10.47	5.88	4.09	2.28
SW-EtOAc	7.85	5.52	4.13	2.25
SW-EtOH	10.70	5.95	4.18	2.29
SW-MeOH	10.33	5.94	4.25	2.28
SW-Acetone	11.01	5.99	4.27	2.26
SW-Insoluble	10.47	6.36	4.37	2.29

sample	*D*_1 max_ (Å)	*D*_2 max_ (Å)	*D*_3 max_ (Å)	*D*_II order 3_ (Å)
DAC-SW-Initial	12.63	7.04	4.11	2,51
DAC-SW-EtOAc	12.51	6.85	4.14	2.17
DAC-SW-EtOH	13.25	7.07	4.14	2.40
DAC-SW-MeOH	N/A			
DAC-SW-Acetone	12.80	6.95	4.17	2.30
DAC-SW-Insoluble	12.23	6.90	4.18	2.28

sample	*D*_1 max_ (Å)	*D*_2 max_ (Å)	*D*_3 max_ (Å)	*D*_4 max_ (Å)	*D*_II order 3_ (Å)	*D*_II order 4_ (Å)
T3-DAC-SW-Initial	16.17	6.56	4.35	3.39	2.24	1.76
T3-DAC-SW-EtOAc	14.62	6.74	4.42	3.52	2.24	1.85
T3-DAC-SW-EtOH	17.45	7.20	4.44	3.47	2.24	1.85
T3-DAC-SW-MeOH	14.52	5.51	4.32	3.41	2.24	1.76
T3-DAC-SW-Acetone	13.91	6.22	4.52	3.61	2.24	1.76
T3-DAC-SW-Insoluble	14.05	6.76	4.66	3.67	2.24	1.78

a The standard deviation for all samples was <0.15 Å (except for T3-DAC-SW-Initial and T3-DAC-SW-MeOH, where the standard deviation was 0.5 Å for *D*_2 max_).

In Table S10, the relative content of each contribution for the retrieved lignin fractions was calculated. It is seen that π–π sandwich structures are the dominant features within the lignin samples. Before allylation, the content of π–π sandwich stacking interactions (*D*_3max_ = 4.09–4.37 Å) was between 75 and 87%, and after allylation (*D*_3max_ = 4.11–4.18 Å), it decreased to 61 and 74%. This suggests that the introduction of allyl functionalities weakens the π–π stacking interactions, leading to the expansion of the lignin aromatic ring interaction distances. As a result, DAC-SW-Lignin has a higher content of T-shaped π–π stacking interactions, and the distances of this signal are shifted to 0.54–1.33 Å. This indicates a more open and accessible structure for these samples, as schematically described in Scheme S3.

*D*_1_ represents a longer distance, which we propose to be related to a lignin molecule superstructure with repeating features/sizes. These structures could be either intra- or intermolecularly formed. It has previously been proposed that these features can be found in native lignin structures.^75^ The shift in *D*_1_ toward a longer distance for the allylated lignin coincides with an increase in the *D*_2_ distance. This implies that the OH groups are situated preferentially on the “edge” of the “sandwiched” structures, which are retained after allylation.

Looking at the data for *D*_1_ in the thermoset samples, it is seen that this distance increases even further. This increase coincides with the appearance of a new signal *D*_4_ corresponding to a distance/size of ≈3.5 Å, not observed in previous studies. The thermoset films contain approximately 45 wt % thiol monomer, and it is reasonable to assume that the *D*_4_ signal relates to a thioether organized structure. It is also seen in Table S11 that the relative signal strength changes with a decrease of the sandwich π–π structures.

### Mechanical Properties of the Thermosets

**Figure 4:**
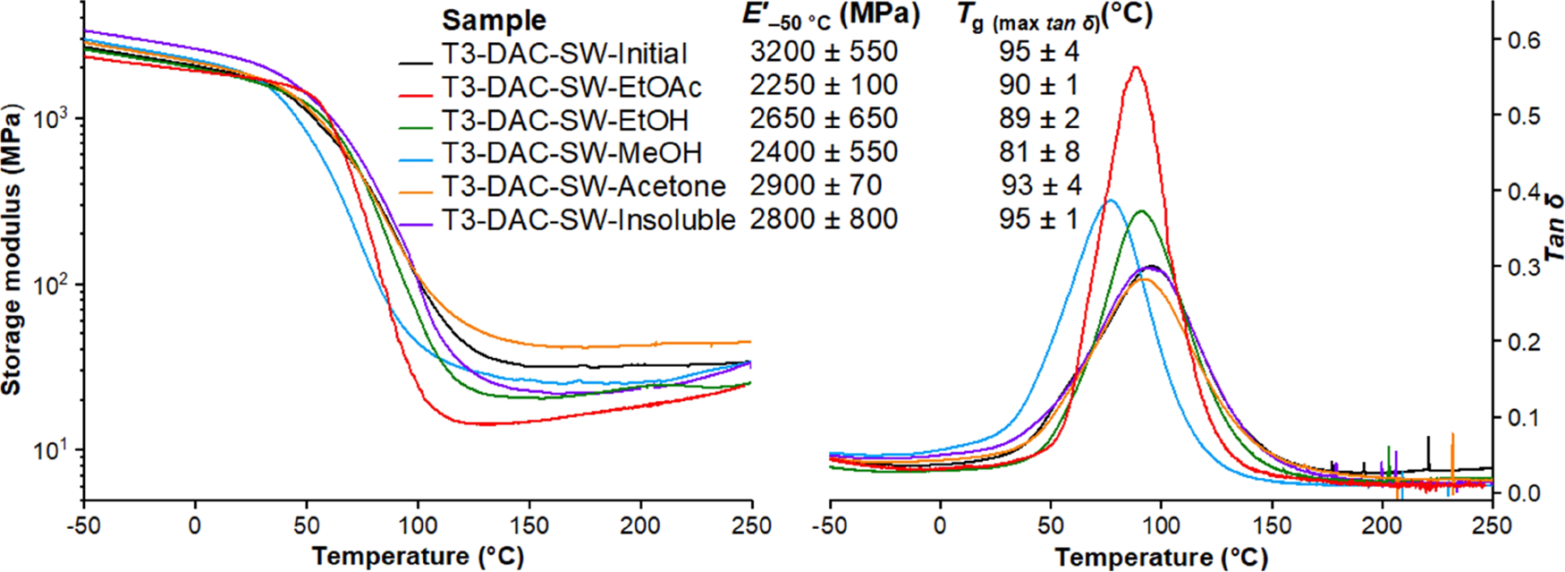
Representative DMA curves of different lignin-based thermosets. Storage modulus (*E*′) is shown on the left and tan δ on the right.

The mechanical properties of conventional thermosets depend on the cross-link density, polarity, and rigidity of the polymer. Rigidity is often introduced by using aromatic monomers that are evenly distributed throughout the cross-linked network as single aromatic entities. These lignin-based thermosets have a different distribution of aromatics, which are more interconnected in larger aggregates. The mechanical properties of the different thermosets in the present study, determined by dynamic mechanical analysis (DMA), all fall in the same range, i.e., there are only minor differences in *T*_g_ (ranged between 81 and 95 °C) and *E*′ (ranged between 2.2 and 3.2 GPa), as shown in Figures 4 and S25. The more narrow and higher tan δ peak for the thermoset based on DAC-SW-EtOAc implies a more homogeneous structure of the network, which can be related to the low initial molecular weight of this fraction. This in combination with a previous discussion on the guaiacyl content shows that the glass transition range is affected by several factors in combination. The small differences in *T*_g_ are minute in comparison to previous studies with less functionalized lignin fractions, where the *T*_g_ of the thermosets comprised between 120 and 157 °C.^46^ The cross-link density is, in general, higher in the present study compared to previous work, as seen for the modulus in the rubbery region, which increases with approximately one decade. There are also differences between the thermosets within the present study with respect to cross-link density. Since all thermosets have approximately the same amount of thiol cross-linker (Table S1), these differences can be related to the lignin structure, although exact details are not known. Comparing the present study with previously reported lignin thiol–ene systems, it is seen that the *T*_g_ is overall significantly lower in the present study.^46^ The structural differences compared to the previous study are that the total allyl functionality is higher (comprised between ≈5.5 and 6.5 mmol/g lignin), the thiol monomer content is higher (comprised between ≈42 and 47%), and the polarity related to hydroxyl groups is lower in the present study. Only the phenolic groups were allylated in the previous study, leaving more unreacted OH groups in the final thermoset. Thioether bonds are more flexible than C–C bonds; hence, a low *T*_g_ is often found for thiol–ene systems.^47^ This indicates that the thioether content, which is directly related to the allyl content, strongly affects the performance of the thermosets. These structural differences not only gave a lower cross-link density but also a higher lignin content and polarity. This clearly shows the importance of lignin content and polarity for the final thermoset properties. The results on mechanical properties also corroborate with the obtained WAXS data on the cured thermosets, where a new signal D_4_ (related to the thioether structure) was found. These structures strongly affect the mechanical performance of the thermosets.

The mechanical performance of lignin-based thermosets was also evaluated using uniaxial tensile testing to learn about the effect on ductility, and the results are summarized in Table S12. As seen on representative stress–strain curves presented in Figure S26, these thermosets exhibit higher elongation at break, between 2.5 and 5.8%, compared to ≈1.5% obtained in the previous study and lower stress at break, between 19 and 49 MPa, compared to ≈68 ± 6 MPa. The results showed also lower Young’s modulus, *E*, between 1.4 and 2.3 GPa compared to ≈4.8 ± 1.5 GPa.^46^ These differences also indicate that the thioether linkages strongly affect the performance as previously discussed. The thermal stability for all thermosets is reasonably good and showed no degradation up to 200 °C (Figure S27). The present thermosets have potential application as adhesives or as composite matrices where the main focus is on mechanical performance and appearance is of less importance.

## Conclusions

The present study on thermosets based on allylated lignin fractions reveals how the structure of the initial technical lignin, its fractions, and the degree of functionalization affect the morphology before and after cross-linking as well as the final mechanical properties. It is shown that the presence of polar moieties, the lignin backbone structure, and the cross-link density are all important, on the molecular level, to determine the morphology on different length scales. The high allyl functionalities and low amounts of polar groups, e.g., OH groups, result in final thermoset polymers where the properties are strongly affected by the thioether linkages. This is also seen in the WAXS data, where a new distance appears in the thermosets. This is in contrast to the properties of lignin-based thermosets with a lower degree of allylation where only the phenolic OH groups were functionalized. Those thermosets exhibited a significantly higher property dependence related to the initial lignin structure. It can be concluded that technical lignin-based thermosets all exhibit a structural ordering on different length scales, ranging from ≈3.5–17.5 Å as determined by WAXS measurements. These ordered structures vary and depend on the initial lignin structure as well as how the lignin has been functionalized and cross-linked.

## Supporting Information Available

Additional experimental details; SEC, ^1^H NMR, ^31^P NMR, FTIR, DSC, and TGA data of lignin fractions before and after modification; RT-FTIR data for the thermal curing of lignin-based thermoset formulations; WAXS plots and the corresponding fits, distances, and contents of repeating features within lignin-based thermosets; and tensile stress–strain and TGA data for lignin-based thermosets (PDF)
