# Supplementary material for: Effect of Molecular Organization on the Properties of Fractionated Lignin-Based Thiol–Ene Thermoset Materials
Source: ACS Omega. 2023 Jul 3;8(28):25478–86. doi: 10.1021/acsomega.3c03022 (PMC10357541; doi:10.1021/acsomega.3c03022)
Supplement: Supplementary file 1 — ao3c03022_si_001.pdf [file ao3c03022_si_001.pdf]

SUPPORTING INFORMATION

EFFECT OF MOLECULAR ORGANIZATION ON THE PROPERTIES OF FRACTIONATED LIGNIN-BASED THIOL-ENE THERMOSET MATERIALS

Iuliana Ribca[1,2], Benedikt Sochor[3], Stephan V. Roth[2,3], Martin Lawoko[1,4], Michael A. R. Meier[5, 6], Mats Johansson[1,2,*]

[1] *Wallenberg Wood Science Center (WWSC), Department of Fibre and Polymer Technology, KTH Royal Institute of Technology, Teknikringen 56-58, SE-100 44 Stockholm, Sweden;*

[2] *Division of Coating Technology, Department of Fibre and Polymer Technology, KTH Royal Institute of Technology, Teknikringen 48, SE-100 44 Stockholm, Sweden;*

[3] *Deutsches-Elektronen Synchrotron (DESY), 22607 Hamburg, Germany;*

[4] *Division of Wood Chemistry and Pulp Technology, Department of Fibre and Polymer Technology, KTH Royal Institute of Technology, Teknikringen 56, SE-100 44, Stockholm, Sweden*

[5] *Institute of Organic Chemistry (IOC), Materialwissenschaftliches Zentrum MZE, Karlsruhe Institute of Technology (KIT), Straße am Forum 7, 76131 Karlsruhe, Germany*;

[6] *Institute of Biological and Chemical Systems—Functional Molecular Systems (IBCS-FMS), Karlsruhe Institute of Technology (KIT), Hermann-von-Helmholtz-Platz 1, 76344 Eggenstein-Leopoldshafen, Germany*; orcid.org/0000-0002-4448-5279

*Corresponding Author, e-mail: matskg@kth.se

- Number of pages: 31
- Number of figures: 27
- Number of schemes: 3
- Number of tables: 12

Table of Contents

Scheme S1. Experimental flow chart of all steps. 4
Procedures 5
Initial Purification of Technical Lignin Powder 5
Lignin Extraction Using Sequential Solvent Fractionation Approach 5
Lignin Allylation 5
Decarboxylation of Allylated Lignin Fractions 6
Preparation of Lignin-Based Thermosets 6
Table S1. Lignin/thiol content in thermosets. 6
Characterization Techniques 6
Size Exclusion Chromatography 6
Proton and Phosphorus Nuclear Magnetic Resonance Spectroscopy 7
Fourier Transform Infrared Spectroscopy (FTIR) and Real Time FTIR 7
Differential Scanning Calorimetry 7
Thermogravimetric Analysis 7
Dynamic Mechanical Analysis 8
Wide-Angle X-ray Scattering 8
Tensile Testing 8
Technical Lignin Fractionation and Allylation 9
Figure S1. DMSO SEC overlay of SW-Lignin fractions. 9
Table S2. M_n, M_m, dispersity, and the yield of SW-Lignin fractions. 9
Figure S2. DMSO SEC overlay of DAC-SW-Lignin fractions. 10
Table S3. M_n, M_m, and dispersity of DAC-SW-Lignin fractions. 10
Table S4. Assignment of the allylic protons in ^{1}H NMR. 11
Figure S3. ^{1}H NMR spectra of SW-Lignin fractions before/after modification. 11
Figure S4. ^{1}H NMR spectra of DAC. 12
Table S5. Percentage of allylated OH functionalities determined by ^{31}P NMR. 12
Figure S5. FTIR spectra of SW-EtOH before and after allylation. 12
Figure S6. FTIR spectra of SW-Lignin fractions before and after allylation. 13
Figure S7. FTIR spectra of DAC. 14
Figure S8. FTIR spectra of TBAB. 14
Figure S9. ^{1}H NMR spectra of commercial and recycled TBAB. 15
Figure S10. DSC thermograms of allylated lignin fractions and T_g. 16
Figure S11. Representative TGA thermograms of allylated lignin fractions. 16

Decarboxylation of Allylated Lignin 17
Scheme S2. Decarboxylation of DAC-SW-Lignin with LiOH. 17
Table S6. The total amount of aliphatic OH and the total amount of allylated aliphatic OH. The amount of etherified and carboxyallylated aliphatic OH. 17
Figure S12. ^{31}P NMR spectra of allylated samples before and after decarboxylation. 18
Figure S13. ^{1}H NMR spectra of allylated and decarboxylated lignin fractions. ...18
Figure S14. FTIR spectra of D-DAC-SW-Lignin...19
Curing Performance of Thermosetting Resins 19
Figure S15. RT-FTIR spectra before curing of T3-DAC-SW-Lignin. 19
Figure S16. RT-FTIR spectra after curing of T3-DAC-SW-Lignin. 20
Table S7. Thiol conversion for different lignin formulations. 20
Figure S17. RT-FTIR spectra for the thermal curing of T3-DAC-SW-EtOH. 21
Morphological Characterization Using X-ray Scattering 21
Figure S18. Summed WAXS pattern for one lignin sample. 22
Figure S19. WAXS signal of SW-Lignin samples. 22
Figure S20. WAXS signal of DAC-SW-Lignin samples. 23
Figure S21. WAXS signal of T3-DAC-SW-Lignin samples. 23
Scheme S3. Schematic description of proposed changes in scattering distances, for lignin fractions after allylation..24
Table S8. Signal's maximum values of the repeating features within SW-Lignin and DAC-SW-Lignin samples. 24
Table S9. Signal's maximum values of the repeating features within T3-DAC-SW-Lignin samples. 24
Figure S22. Fitted WAXS intensity profiles of SW-Lignin samples. 25
Figure S23. Fitted WAXS intensity profiles of DAC-SW-Lignin samples. 26
Figure S24. Fitted WAXS intensity profiles of T3-DAC-SW-Lignin samples. 27
Table S10. Contents of the repeating features within SW-Lignin and DAC-SW-Lignin samples. 28
Table S11. Contents of the repeating features within T3-DAC-SW-Lignin samples. 28
Mechanical Properties of the Thermosets 29
Figure S25. Loss modulus curves of lignin-based thermosets. 29
Table S12. Strain at break, stress at break, and Young's modulus 29
Figure S26. Representative tensile stress-strain curves for the thermosets. 30
Figure S27. Representative TGA curves of different lignin-based thermosets. ..30
References 31

Scheme S1. Experimental flow chart of lignin washing, fractionation, allylation, and thermal curing.

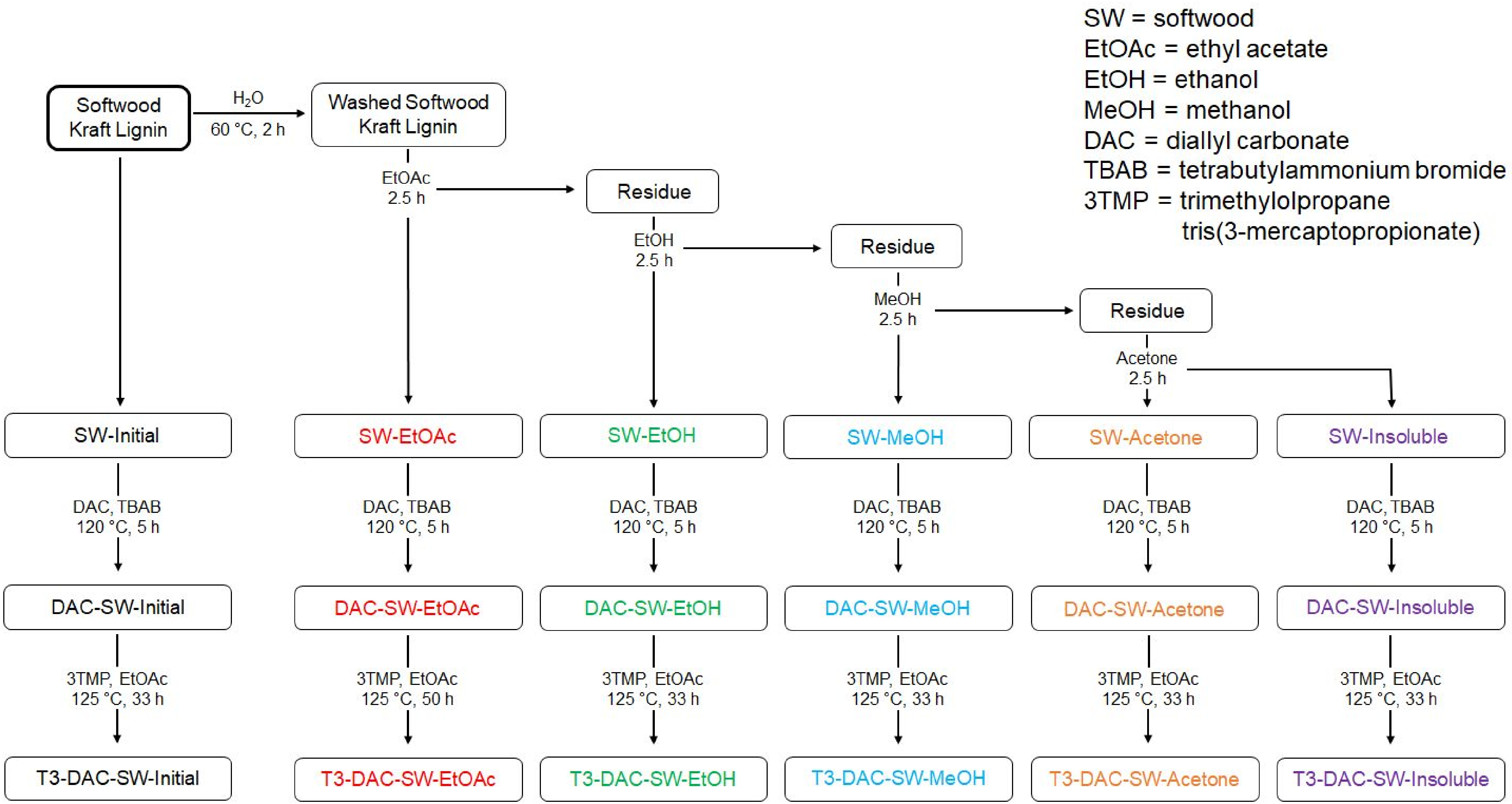

Procedures

Initial Purification of Technical Lignin Powder

The washing step was performed in order to remove various impurities such as inorganics. Approximately 35 g of lignin was dispersed in 700 mL of deionized water and magnetically stirred at 60 °C for 2 h. Subsequently, the lignin was filtered using a sintered funnel (pore size 4). The procedure was repeated until the pH of the filtrate was above 5.5 (2–3 times). The washed lignin (SW-Initial) was dried in a vacuum oven at 50 °C overnight. The recovery yield was calculated to be 93 ± 2%. The washing step was performed ten times.

Lignin Extraction Using Sequential Solvent Fractionation Approach

15 ± 2 g of washed lignin was mixed with 150 ± 20 mL of EtOAc and magnetically stirred at room temperature for 2 h. The insoluble lignin was separated from the mixture by filtration through a filter paper (grade 3 Munktell) and subsequently dried in a vacuum oven at 50 °C overnight. The EtOAc solution was then rotary evaporated to recover the EtOAc soluble fraction. The EtOAc soluble lignin was then dried in a vacuum oven at 50 °C overnight and subsequently re-dissolved in acetone, precipitated in 200 mL of deionized water and finally freeze-dried. A fine, brown powder was obtained as the final product. The same procedure was then applied to the insoluble fraction using an extraction sequence of firstly EtOH, secondly MeOH, and finally acetone as extracting solvents. The lignin to solvent ratio was 1:10 (g/mL) in all cases. Five different fractions were obtained and named accordingly: SW-EtOAc, SW-EtOH, SW-MeOH, SW-Acetone, and SW-Insoluble. The last fraction was denoted as SW-Insoluble because it was not soluble in the four previous mentioned solvents (EtOAc, EtOH, MeOH, and acetone), however it is soluble in DMSO, which enables its characterization.[1, 2] The extraction step was performed four times. The fractionation yields are reported in Table S2.

Lignin Allylation

The initial lignin (SW-Initial) and all five fractions (1 g of each, ≈6.5 mmol of the different OH groups/g lignin) were respectively placed in different 10 mL pressure vials. Diallyl carbonate (3 eq. per hydroxyl group, ≈2.8 mg) and tetrabutylammonium bromide (1 eq. per hydroxyl group, 2.1 mg) were added and the pressure vials were then sealed. For each lignin fraction, the exact content of all different OH groups was calculated, and the DAC and TBAB content was adjusted accordingly. The mixture was magnetically stirred at 120 °C for 5 h. The reaction mixture was then left to cool to room temperature and then dissolved in 50 mL of EtOAc. The organic phase was extracted with deionized water to remove TBAB. The catalyst was then recovered from the aqueous mixture by rotary evaporating water and finally dried in a vacuum oven to give a pale-yellow solid (with a yield of 85%). The organic phase was dried over $MgSO_4$, filtered, and then concentrated to a total volume of approximately 5 mL using a rotary evaporator. The concentrated solution was precipitated in 200 mL ice-cold cyclohexane under vigorous magnetic stirring. Chemically modified lignin was filtered by using filter paper (grade 3, Munktell) and washed several times with cyclohexane. Allylated lignin was then dried in a vacuum oven at 50 °C overnight to give a bright-yellow powder.[3] The samples were denoted DAC-SW-Initial, DAC-SW-Solvent

(solvent = EtOAc, EtOH, MeOH, Acetone), and DAC-SW-Insoluble. The lignin allylation was performed at least two times for each fraction.

Decarboxylation of Allylated Lignin Fractions

Approximately 100 mg of allylated lignin (with ≈6.5 mmol of the different OH groups before allylation/g lignin) was dissolved in 1 mL of tetrahydrofuran (THF) in a vial. For each lignin fraction, the exact content of all different OH groups before allylation was considered, and the LiOH content was adjusted accordingly (for 1 mmol of the different OH it was added approximatively 100 mg of LiOH). Lithium hydroxide (65.0 mg, 2.7 mmol) was dissolved in 1 mL of H_2O, mixed with the dissolved lignin, and then stirred at room temperature for 1 week. The product was then precipitated by adding 1 M HCl (100 mL) and filtered. The product was then washed with 1 M HCl (50 mL), water (100 mL), and dried in a vacuum oven at 50 °C overnight.[3] The decarboxylated lignin was obtained as a yellow powder in yields of 67 ± 14 wt%. The obtained samples were denoted D-DAC-SW-Initial, D-DAC-SW-Solvent (solvent = EtOAc, EtOH, MeOH, Acetone), and D-DAC-SW-Insoluble.

Preparation of Lignin-Based Thermosets

For each free-standing sample, (40 ± 5) mg of allylated lignin was mixed with the thiol cross-linker in a vial. The equivalent ratio between the reactive groups (ene:thiol) was kept at 1:1. 150 mL of EtOAc were then added, to ensure a better mixing. The obtained mixtures were poured into soft silicone molds and left in the fume hood for several hours in order to enable the solvent to evaporate and form a smooth film. The molds were then transferred into an oven and cured at 125 °C for 33 h (the resin prepared from DAC-SW-EtOAc was cured at 125 °C for 50 h). The obtained thermosets were removed from the mold directly after they were taken out of the oven.[4] All formulations and thermosets were denoted in the following way: T3-DAC-SW-Initial, T3-DAC-SW-Solvent (solvent = EtOAc, EtOH, MeOH, Acetone), T3-DAC-SW-Insoluble. At least five samples were prepared from each allylated lignin fraction.

Table S1. Lignin/thiol content in thermosets.

Sample	Wt % allylated lignin	Wt % thiol
T3-DAC-SW-Initial	53.0 ± 0.7	47.0 ± 0.7
T3-DAC-SW-EtOAc	53.8 ± 0.4	46.2 ± 0.4
T3-DAC-SW-EtOH	54.3 ± 0.1	45.7 ± 0.1
T3-DAC-SW-MeOH	53.6 ± 0.7	46.4 ± 0.7
T3-DAC-SW-Acetone	55.2 ± 0.7	44.8 ± 0.7
T3-DAC-SW-Insoluble	58.2 ± 1.3	41.8 ± 1.3

Characterization Techniques

Size Exclusion Chromatography (SEC)

Size exclusion chromatography was used to determine the lignin number-average molar mass (M_n), weight-average molar mass (M_w), and dispersity ($Đ$). Prior to measurement, all samples were completely dissolved in the elution solvent (5 mg/mL) and filtered through a syringe filter of 0.45 µm pore size. The elution solvent was

DMSO with 0.5% (w/w) lithium bromide (LiBr) at a flow rate of 0.5 mL/min. The measurements were done by using SECcurity 1260 infinity GPC System equipped with a refractive index (RI) detector, a PSS GRAM precolumn, two PSS GRAM separation columns (particle size 10 µm and pore size 100 and 10000 Å, with dimensions 300 mm × 8 mm), at 60 °C. Pullulan standards were used as an internal standard with a molar mass range of 342–708000 g/mol. The SEC data should thus be considered as trends, rather than absolute values because of the lack of calibration standards with high structural similarity to technical lignin molecules. All data represent an average of two to six measurements.

Proton and Phosphorus Nuclear Magnetic Resonance Spectroscopy (^{1}H and ^{31}P NMR)

All spectra were recorded on a Bruker Avance III HD 400 MHz instrument with a BBFO probe equipment with a Z-gradient coil and processed as described previously.[2] Integration regions used for lignin in ^{31}P NMR spectrum were: aliphatic OH (149.5–145.5 ppm), C_5-substituted OH (144.7–140.1 ppm), guaiacyl OH (140.1–138.8 ppm), p-hydroxyphenyl OH (138.8–137.0 ppm), carboxylic acid OH (136.0–133.6 ppm).[5] All individual data represent an average of two to four measurements.

Fourier Transform Infrared Spectroscopy (FTIR) and Real Time FTIR (RT-FTIR)

PerkinElmer Spectrum 100 instrument was used to analyze the chemical composition of the reagents and products. Attenuated total reflection (ATR) mode with a diamond crystal was used. The instrument was equipped with a temperature control unit (Specac, Heated Golden Gate Controller). All FTIR spectra were acquired at room temperature. For each sample, 16 scans were done, with a resolution of 4 cm^{-1} between 4000 and 600 cm^{-1}. For RT-FTIR, a new scan was acquired every minute with a resolution of 4 cm^{-1}. Thiol conversion was calculated by monitoring the absorption signal of S-H bond at 2607–2533 cm^{-1}. RT-FTIR studies were performed at 125 °C. FTIR spectra were analyzed with Spectrum software and RT-FTIR spectra with TimeBase® software from Perkin-Elmer. FTIR spectra were baseline corrected and normalized to the 1509 cm^{-1} signal corresponding to the aromatic C=C stretching.[6]

Differential Scanning Calorimetry (DSC)

Mettler Toledo DSC1 was used to analyze the thermal transitions of lignin samples. 10 mg of each sample were placed in a 100 µL aluminum crucible and covered with a pierced lid. The samples were then heated to 105 °C and held for 20 min, then cooled down to 20 °C and held for 10 min and finally heated up to 200 °C. The heating/cooling rate was 10 °C/min. The second heating midpoint was used to determine the glass transition temperature (T_g). The DAC-SW-EtOAc sample was cooled to –80 °C. All individual data represent an average of three to six measurements.

Thermogravimetric Analysis (TGA)

Mettler Toledo TGA/DSC1 instrument was used to study the thermal behavior of the samples. 10 mg of each sample were placed in a ceramic crucible for the analysis. For the first 10 minutes, samples were kept at 30 °C, afterwards they were heated up

to 800 °C, with a rate of 5 °C/min (in a nitrogen atmosphere, flow rate of 50 mL/min) and then kept at 800 °C for 10 min.

Dynamic Mechanical Analysis (DMA)

DMA Q800 instrument was used to investigate the viscoelastic properties of the thermosets in tensile mode. Samples were cooled to –100 °C and held for 5 min (using liquid nitrogen). The samples were then heated to 250 °C with a heating rate of 3 °C/min. The preload force of 0.01 N (force track 125%), with a strain of 0.1% at 1 Hz was used to measure the free sanding samples (12 mm × 5.2 mm × 0.15 mm). Storage modulus (E'), loss modulus (E''), and *tan δ* were investigated. The maximum of *tan δ* was used to calculate the T_g of the thermosets. All individual data represent an average of two to three measurements.

Wide-Angle X-ray Scattering (WAXS)

Using wide-angle X-ray scattering the molecular structure of materials can be studied. All X-ray experiments were performed at the beamline P03 at PETRA III (Hamburg, Germany). The details on the experimental set-up and procedure for data reduction as well as analysis can be found on page SI20. The powder samples (SW-Lignin, DAC-SW-Lignin), and the films (T3-DAC-SW-Lignin) were mounted in custom-made 3D-printed sample holders. The pellet pressing die and the hydraulic laboratory press were used to create pellets of lignin powder samples, with a diameter of 13 mm and a thickness of 1 mm. The geometry of the lignin based-thermosets was 12 mm × 5.2 mm × 0.15 mm.

Uniaxial Tensile Testing

Instron 5944 instrument (equipped with a 500 N cell) was used to determine the tensile properties of the lignin-based thermosets. The tensile grip distance was 15 mm and the crosshead speed was set to 5 mm/min. Rectangular samples (with approximate dimensions of 15 mm × 5.2 mm × 0.15 mm) were conditioned for 1 week in an environment controlled room where also the measurements were made (temperature of 22 °C and relative humidity of 50 ± 2%). All individual data represent an average of three to nine measurements.

Technical Lignin Fractionation and Allylation

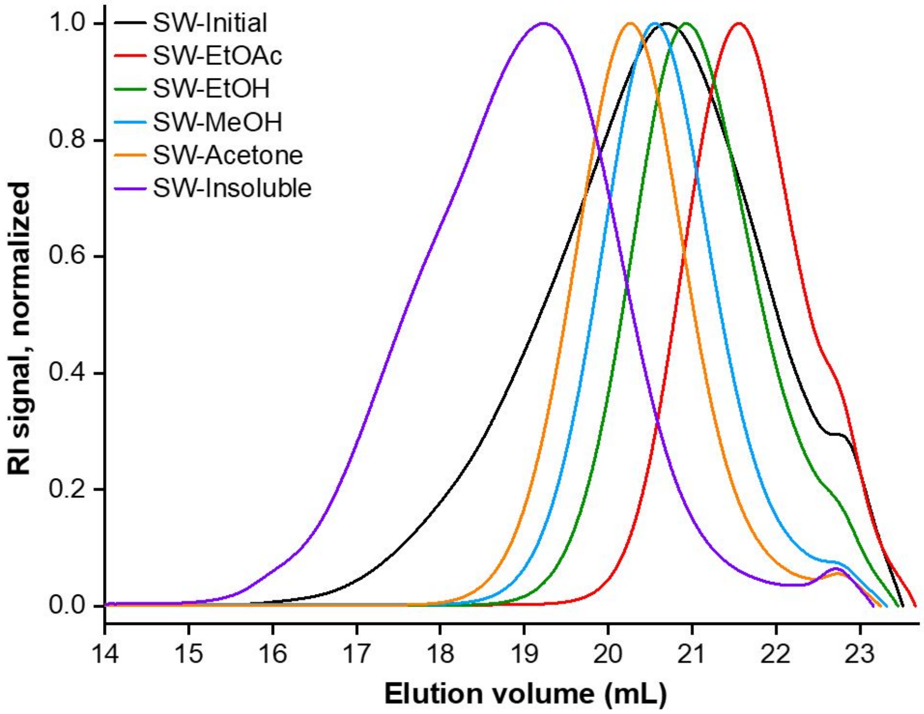

Figure S1. DMSO SEC overlay of SW-Lignin fractions. Pullulan standards were used as an internal standard.

Table S2. Number/Weight average molar mass, dispersity (determined by SEC), and also the yield of SW-Lignin fractions.

Sample	M_n (g/mol)	M_w (g/mol)	*Đ*	Yield (%)
SW-Initial	2050 ± 50	6500 ± 100	3.2 ± 0.1	-
SW-EtOAc	850 ± 50	1250 ± 50	1.5 ± 0.1	23 ± 1
SW-EtOH	1300 ± 300	2150 ± 300	1.9 ± 0.4	22 ± 1
SW-MeOH	2000 ± 500	3250 ± 650	1.6 ± 0.1	8 ± 1
SW-Acetone	2850 ± 600	4850 ± 950	1.7 ± 0.1	10 ± 1
SW-Insoluble	7950 ± 1900	27050 ± 5150	3.4 ± 0.2	34 ± 1

*Pullulan standards were used as an internal standard. The SEC data should be considered as trends, rather than absolute values

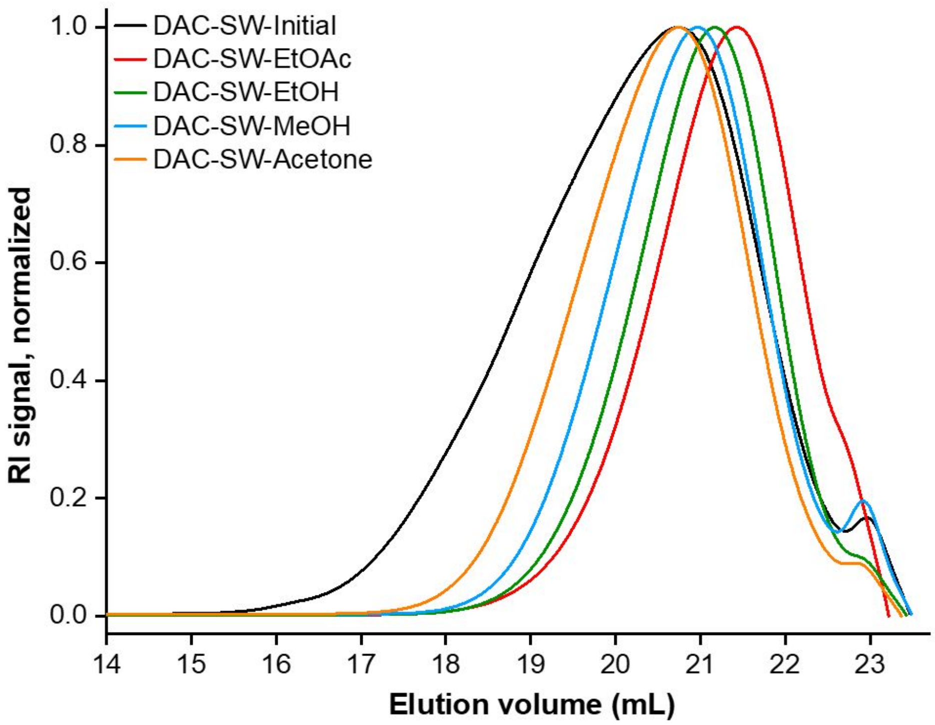

Figure S2. DMSO SEC overlay of DAC-SW-Lignin fractions. Pullulan standards were used as an internal standard.

Table S3. Number/Weight average molar mass and dispersity of DAC-SW-Lignin fractions determined by SEC.

Sample	M_n (g/mol)	M_w (g/mol)	$Đ$
DAC-SW-Initial	2100 ± 300	9800 ± 800	2.9 ±1.6
DAC-SW-EtOAc	1050	1950	1.8
DAC-SW-EtOH	1050 ± 200	2100 ± 400	2.7 ± 1.0
DAC-SW-MeOH	1500 ± 200	3600 ± 500	2.4 ± 0.3
DAC-SW-Acetone	1900 ± 200	5000 ± 750	2.6 ± 0.3
DAC-SW-Insoluble		N/A	

*Pullulan standards were used as an internal standard. The SEC data should thus be considered as trends, rather than absolute values

Table S4. Assignment of the allylic protons in ^{1}H NMR.

Allylic protons	δ (ppm)
2	6.28–5.53
3	5.53–4.76
1	4.76–4.07

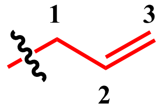

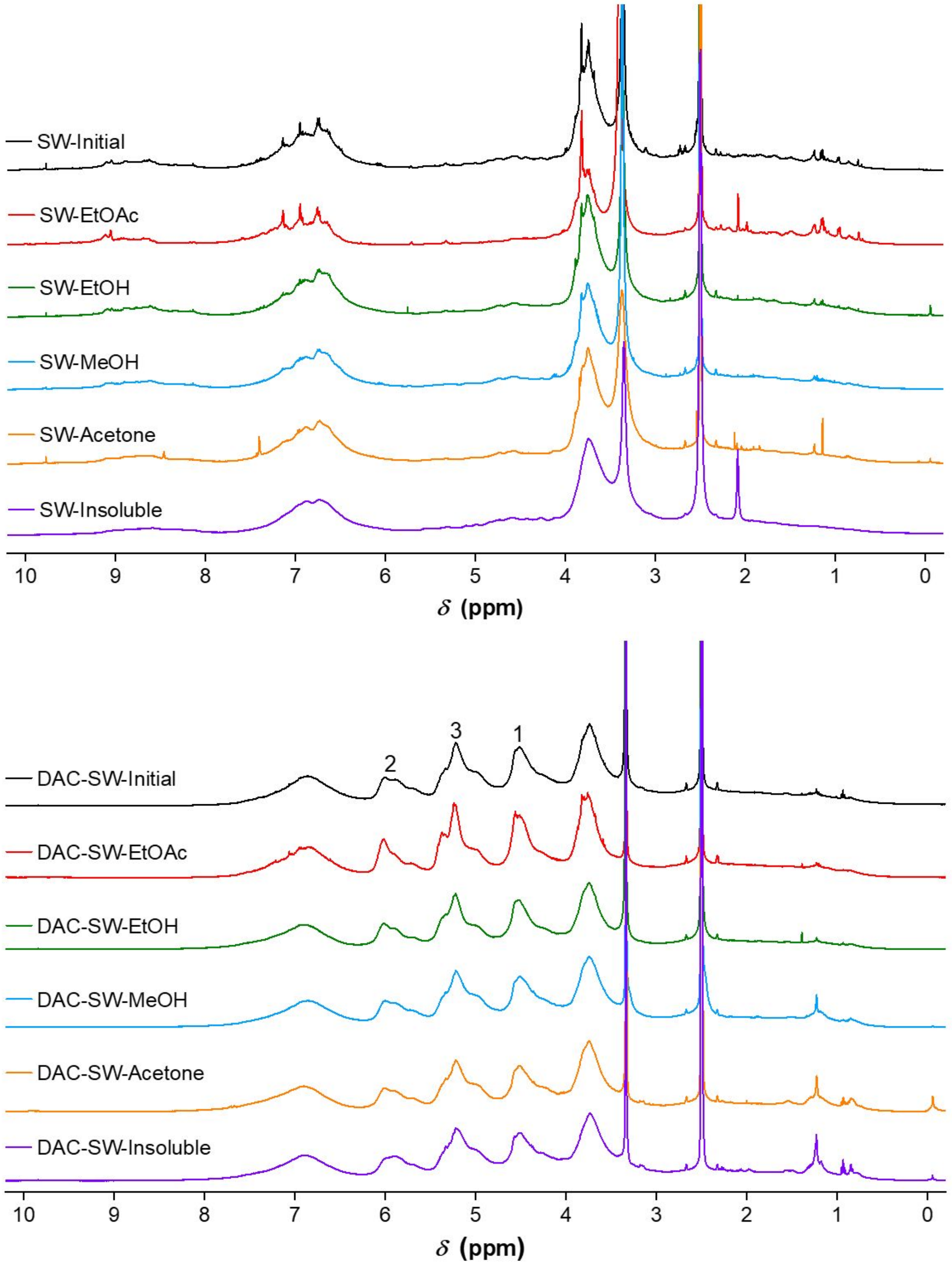

Figure S3. ^{1}H NMR spectra (DMSO-d_6) of SW-Lignin fractions before modification (up) and after modification (bottom). The signals at: δ = 2.08 ppm results from residual acetone, δ = –0.06 ppm results from silicone grease, and at δ = 3.34 ppm results from the water present in DMSO-d_6.

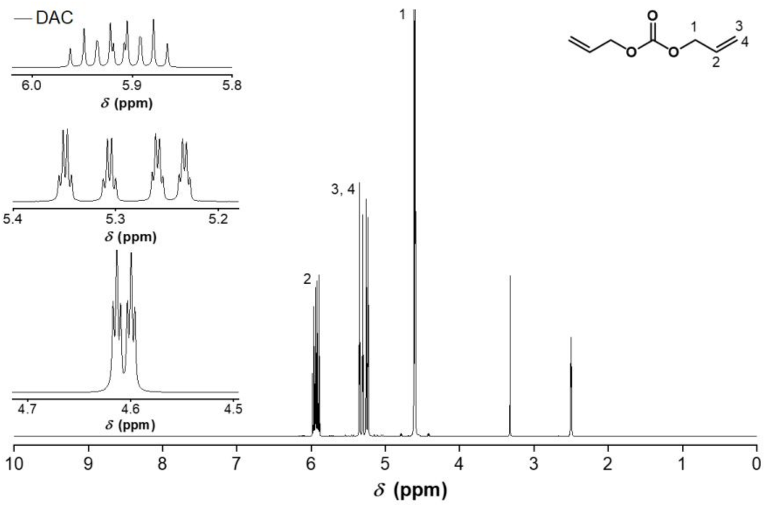

Figure S4. ^1H NMR (DMSO-d_6) spectra of DAC with zoom in the regions of the allyl functionality. The signal at δ = 3.34 ppm results from the water present in DMSO-d_6.

Table S5. Percentage of allylated OH functionalities determined by ^{31}P NMR.

Sample	Conversion of OH groups (%)/Allyl content (mmol/g)				
	**Aliphatic OH**	**C_5-substituted OH**	**Guaiacyl OH**	**p-hydroxyphenyl OH**	**Carboxylic acid OH**
DAC-SW-Initial	68 ± 2/ 1.5 ± 0.1	97 ± 2/ 2.2 ± 0.1	99 ± 1/ 2.1 ± 0.0	96 ± 2/ 0.3 ± 0.0	95 ± 2/ 0.5 ± 0.0
DAC-SW-EtOAc	57 ± 14/ 0.5 ± 0.2	95 ± 1/ 2.0 ± 0.0	99 ± 1/ 2.8 ± 0.0	97 ± 1/ 0.4 ± 0.0	97 ± 3/ 0.6 ± 0.0
DAC-SW-EtOH	65 ± 3/ 1.3 ± 0.1	97 ± 1/ 2.2 ± 0.0	99 ± 1/ 2.0 ± 0.0	97 ± 2/ 0.4 ± 0.0	97 ± 2/ 0.5 ± 0.0
DAC-SW-MeOH	67 ± 8/ 1.5 ± 0.2	96 ± 1/ 2.3 ± 0.0	99 ± 1/ 1.9 ± 0.1	99 ± 1/ 0.4 ± 0.0	96 ± 4/ 0.4 ± 0.0
DAC-SW-Acetone	67 ± 5/ 1.2 ± 0.1	96 ± 1/ 2.4 ± 0.0	99 ± 1/ 1.7 ± 0.0	99 ± 1/ 0.4 ± 0.0	98 ± 1/ 0.4 ± 0.0
DAC-SW-Insoluble	67 ± 4/ 1.8 ± 0.1	96 ± 2/ 1.9 ± 0.1	98 ± 2/ 1.2 ± 0.0	99 ± 1/ 0.3 ± 0.0	93 ± 7/ 0.3 ± 0.0

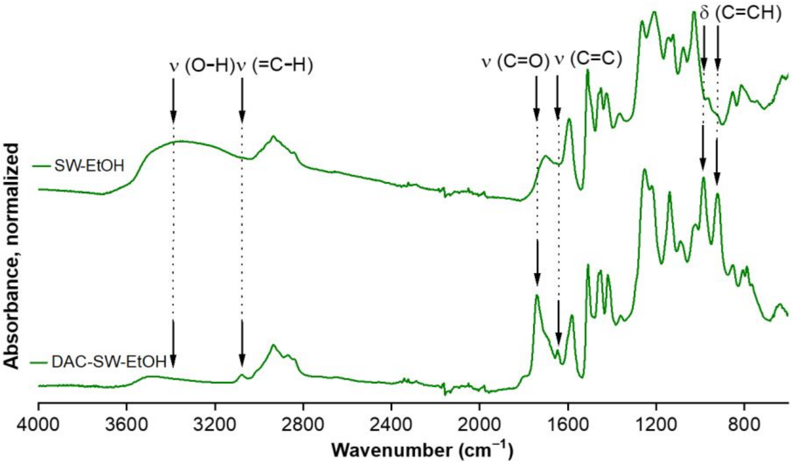

Figure S5. FTIR spectra of SW-EtOH before and after allylation.

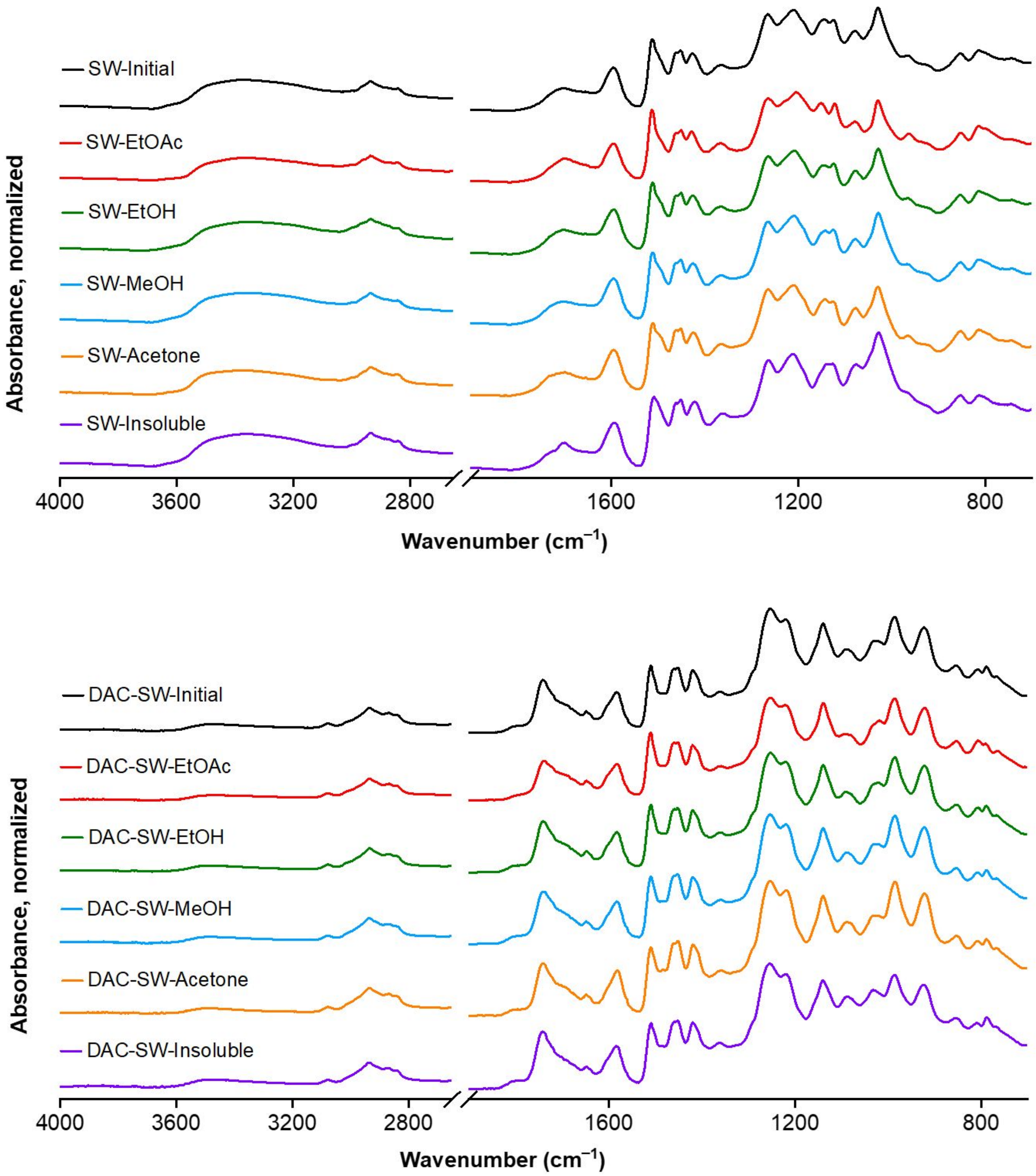

Figure S6. FTIR spectra of SW-Lignin fractions before allylation (up) and after allylation (bottom). The region between 2650–1900 cm^{-1} was omitted due to absorbance of diamond ATR crystal in this region.

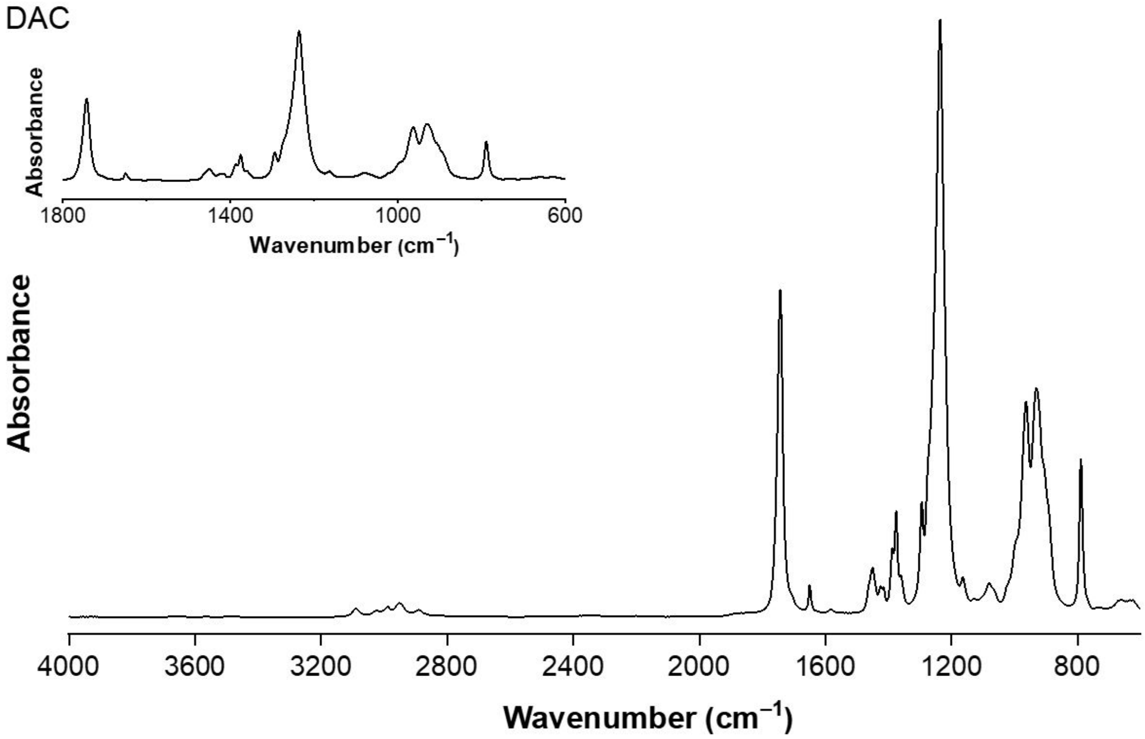

Figure S7. FTIR spectra of DAC, with a zoom of the region 1800–600 cm^{-1}.

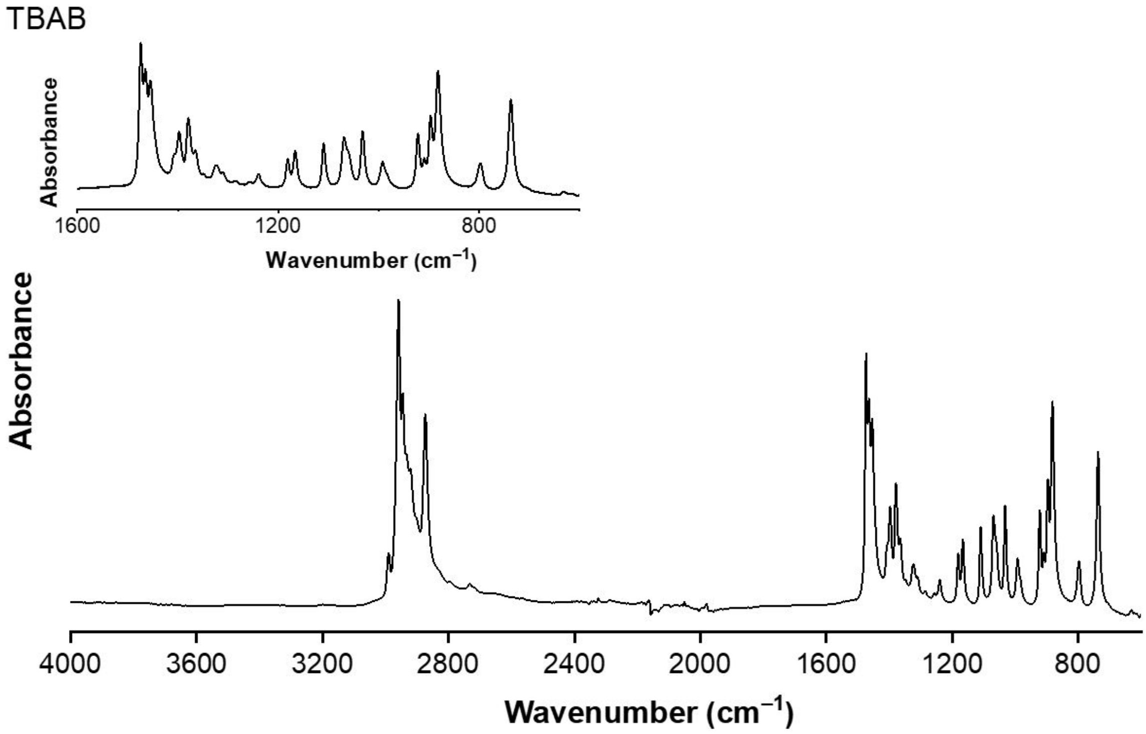

Figure S8. FTIR spectra of TBAB, with a zoom of the region 1600–600 cm^{-1}.

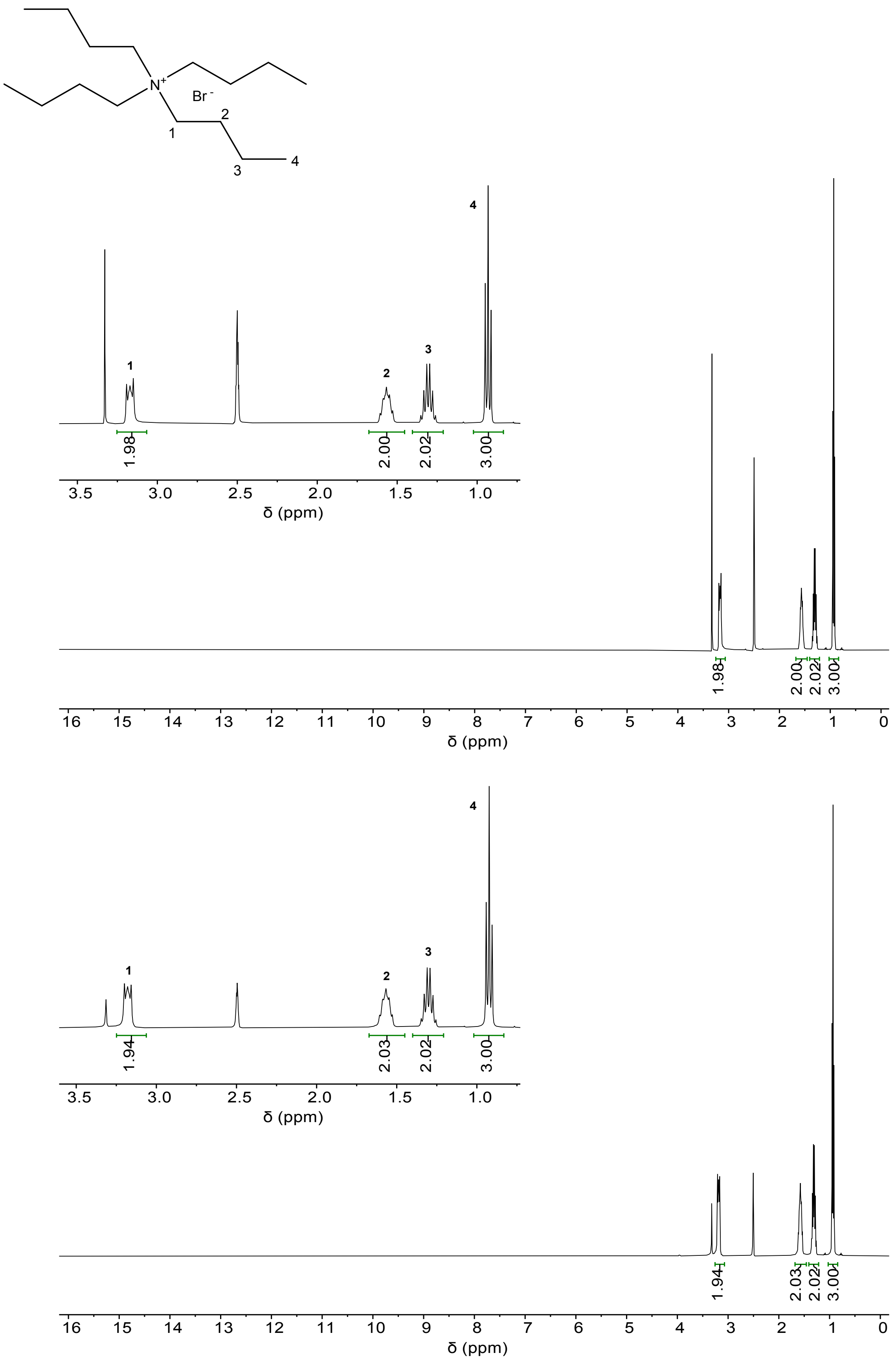

Figure S9. ^{1}H NMR spectra of commercial TBAB (up) and recycled TBAB from DAC-SW-EtOH (bottom) in DMSO-d_6.

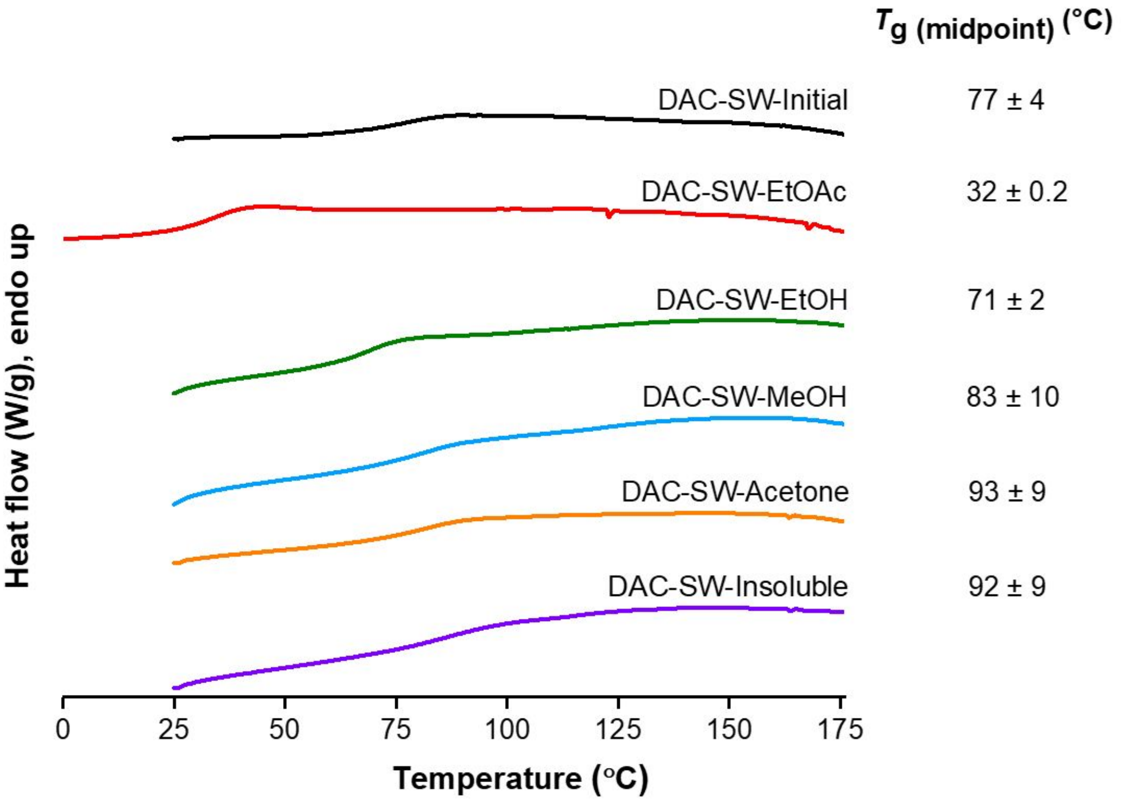

Figure S10. DSC thermograms of allylated lignin fractions and glass transition temperature (T_g, the second heating midpoint).

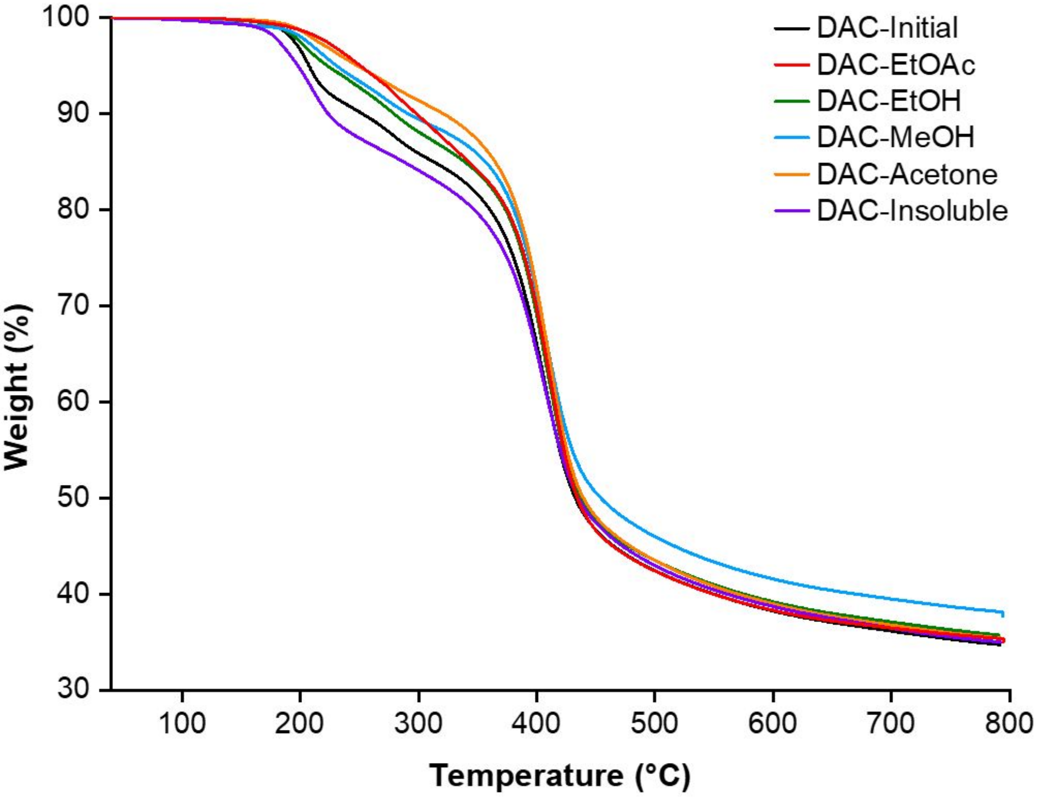

Figure S11. Representative TGA thermograms of allylated lignin fractions.

Decarboxylation of Allylated Lignin

Scheme S2. Decarboxylation of DAC-SW-Lignin with LiOH.

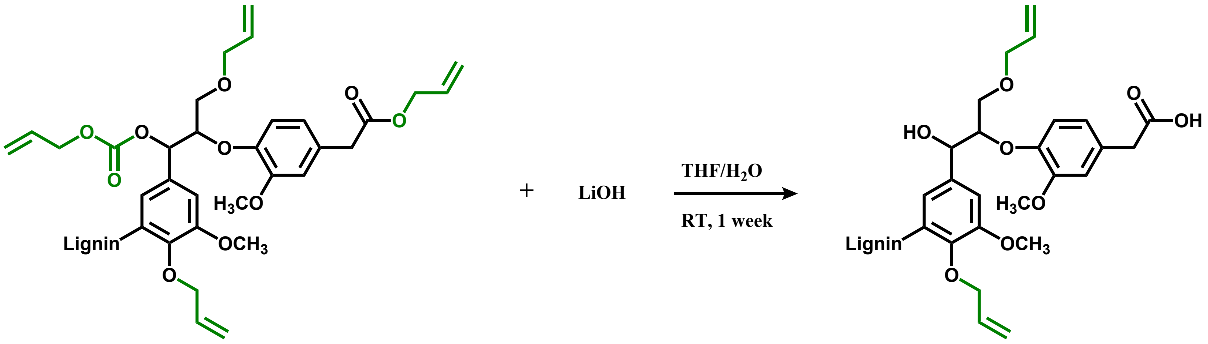

* Decarboxylation reaction was performed in order to determine if hydroxyl groups were etherified or carboxyallylated. From previous studies it was shown that the phenolic OH are etherified and that carboxylic acid OH are most likely to be converted to the corresponding allyl esters.[3, 7] For a better visualization, it was assumed that all aliphatic OH were fully functionalized (carboxyallylated or etherified).

Table S6. The amount of aliphatic OH before allylation (representing 100%, first column) and the total amount of allylated aliphatic OH (second column). The third and fourth columns show the amount/percentage of etherified and carboxyallylated aliphatic OH based on the amount of aliphatic OH before allylation. All data were determined by ^{31}P NMR.

Sample	Aliphatic OH	Allylated Aliphatic OH	Etherified Aliphatic OH	Carboxyallylated Aliphatic OH
SW-Initial	2.27 (100%)			
DAC-SW-Initial		1.47 (65%)		
D-DAC-SW-Initial			0.83 (37%)	0.64 (28%)
SW-EtOAc	0.94 (100%)			
DAC-SW-EtOAc		0.40 (43%)		
D-DAC-SW-EtOAc			0.25 (27%)	0.15 (16%)
SW-EtOH	2.06 (100%)			
DAC-SW-EtOH		1.37 (67%)		
D-DAC-SW-EtOH			0.66 (32%)	0.71 (35%)
SW-MeOH	2.15 (100%)			
DAC-SW-MeOH		1.16 (54%)		
D-DAC-SW-MeOH			1.06 (49%)	0.10 (5%)
SW-Acetone	1.82 (100%)			
DAC-SW-Acetone		1.21 (67%)		
D-DAC-SW-Acetone			0.60 (33%)	0.61 (34%)
SW-Insoluble	2.60 (100%)			
DAC-SW-Insoluble		1.65 (64%)		
D-DAC-SW-Insoluble			0.82 (32%)	0.83 (32%)

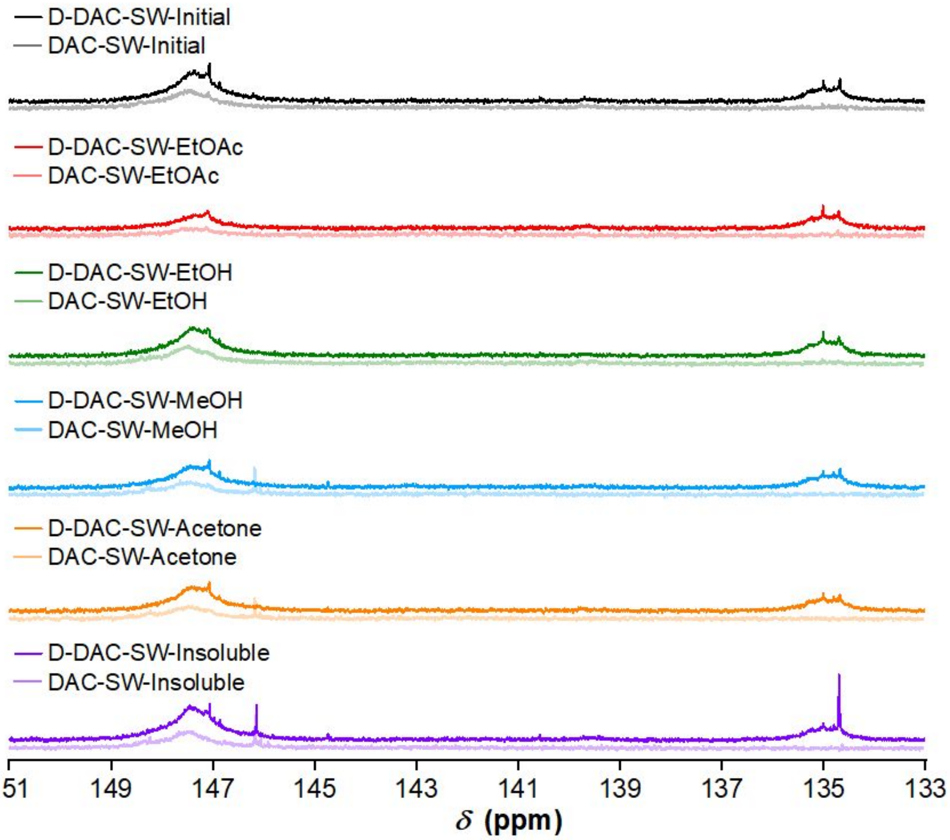

Figure S12. ^{31}P NMR spectra comparison between allylated samples before (DAC-SW-Lignin, bottom) and after decarboxylation (D-DAC-SW-Lignin, up).

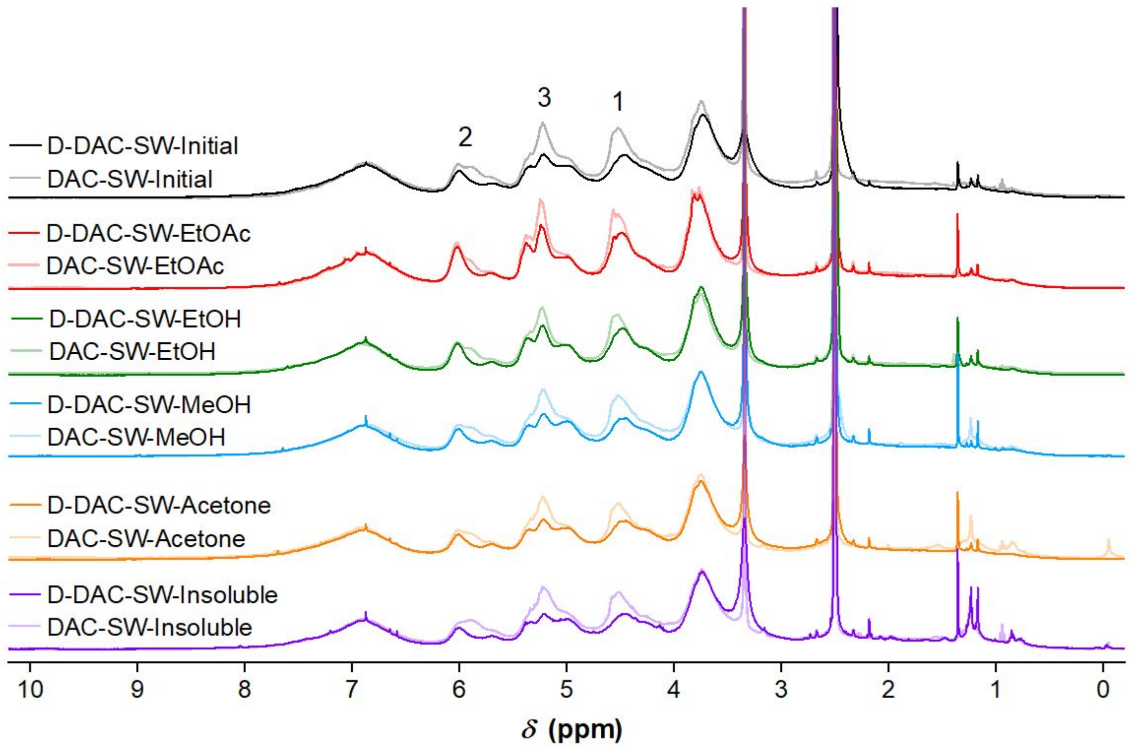

Figure S13. ^{1}H NMR spectra (DMSO-d_6) of DAC-SW-Lignin and D-DAC-SW-Lignin fractions. The signal at δ = 3.34 ppm results from the water present in DMSO-d_6.

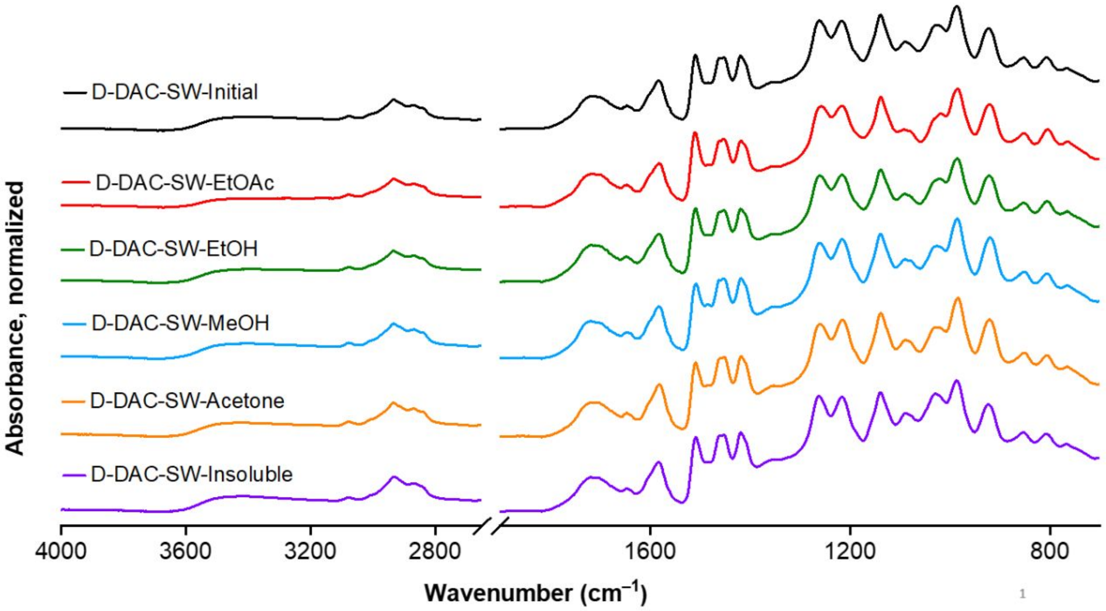

Figure S14. FTIR spectra of D-DAC-SW-Lignin.

Curing Performance of Thermosetting Resins

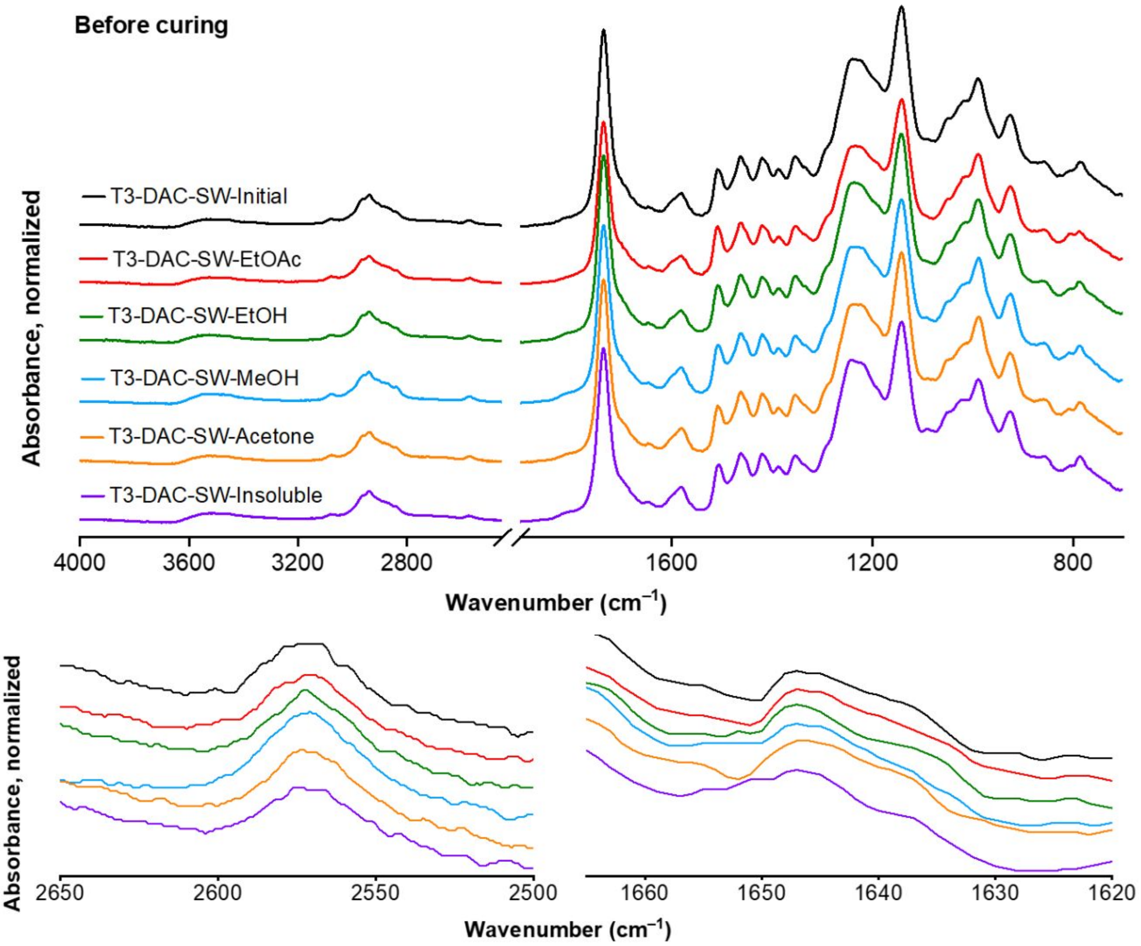

Figure S15. FTIR spectra before curing of T3-DAC-SW-Lignin with zoom in the regions of thiol (2568 cm–1) and ene (1647 cm–1) signals.

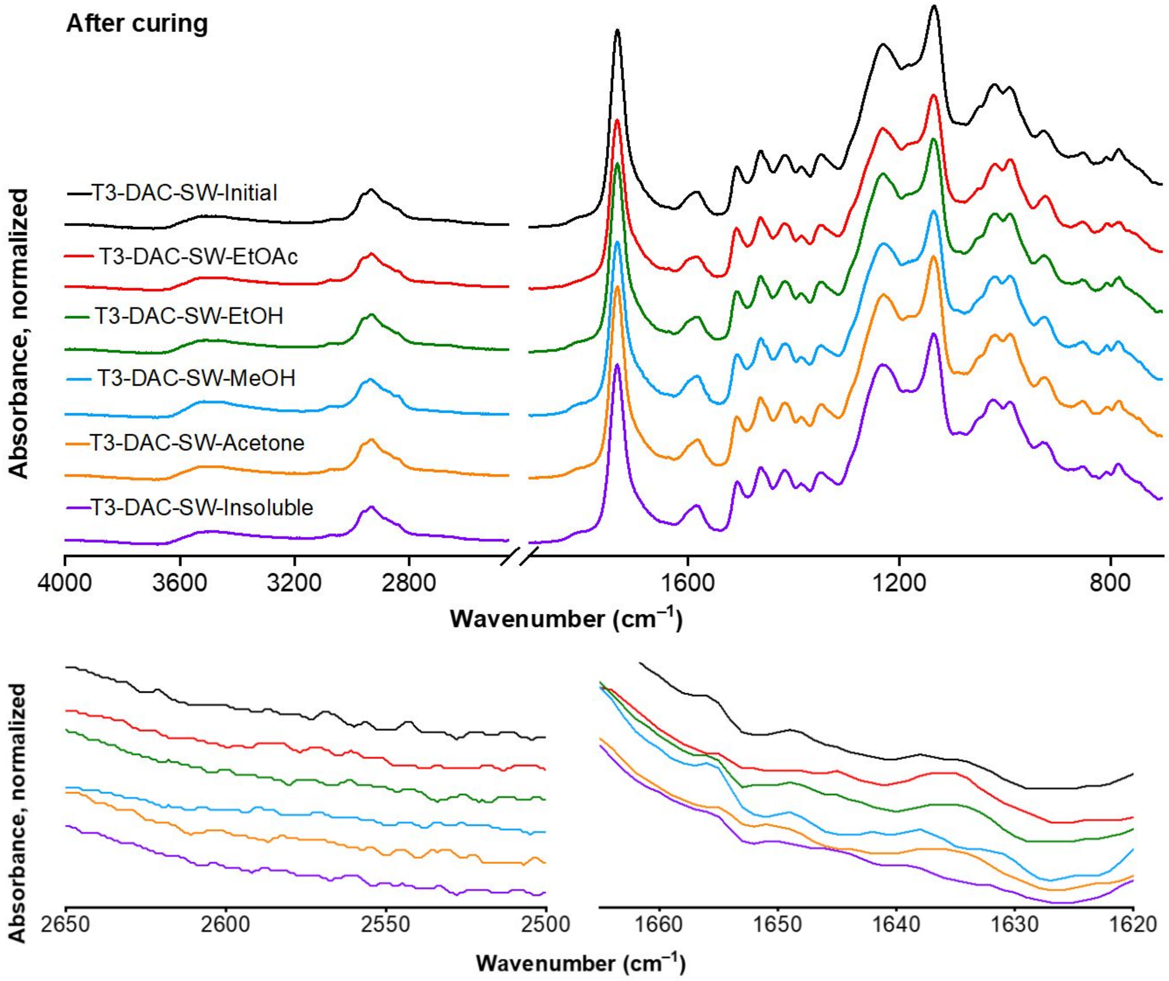

Figure S16. FTIR spectra after curing of T3-DAC-SW-Lignin with zoom in the regions of thiol (2568 cm^{-1}) and ene (1647 cm^{-1}) signals.

Table S7. Thiol conversion versus time as monitored by RT-FTIR for different lignin formulations.

	Thiol conversion				
**Sample**	**20%**	**50%**	**75%**	**95%**	**100%**
T3-DAC-SW-Initial	4.5 h	13.3 h	20.5 h	29.0 h	31.6 h
T3-DAC-SW-EtOAc	7.6 h	20.6 h	30.2 h	41.2 h	48.7 h
T3-DAC-SW-EtOH	5.1 h	14.2 h	21.4 h	29.1 h	32.5 h
T3-DAC-SW-MeOH	2.9 h	8.0 h	13.2 h	21.8 h	29.8 h
T3-DAC-SW-Acetone	3.8 h	10.5 h	16.5 h	23.3 h	25.3 h
T3-DAC-SW-Insoluble	3.1 h	9.0 h	14.2 h	21.4 h	25.0 h

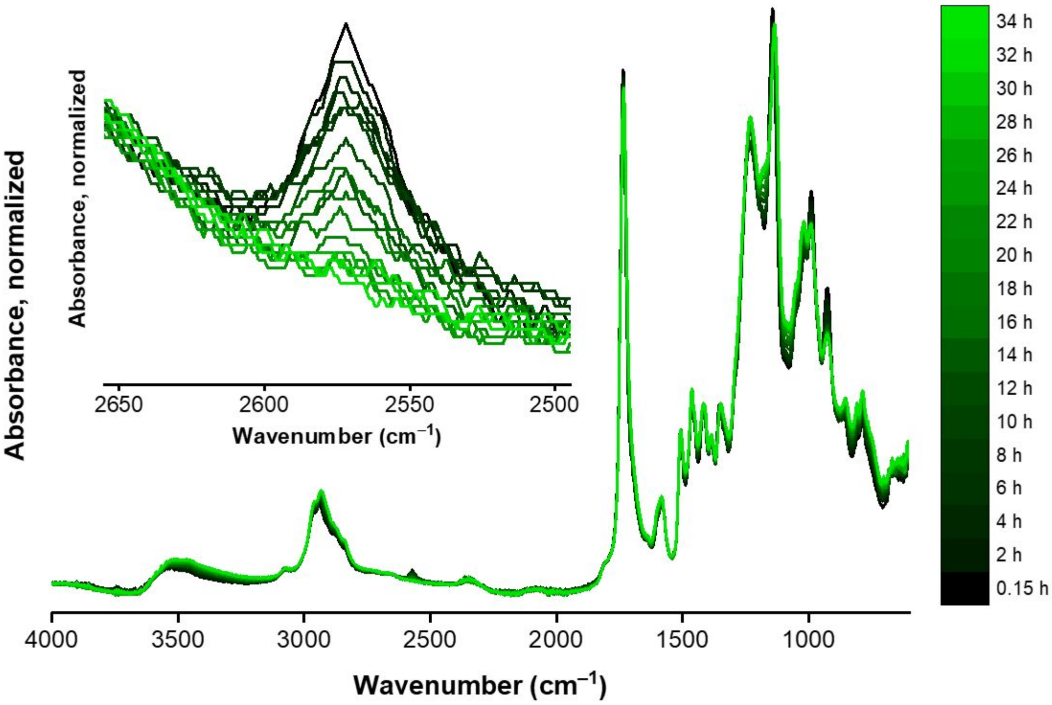

Figure S17. RT-FTIR spectra for the thermal curing of T3-DAC-SW-EtOH at 125 °C with zoom in the region of -SH signal, as a function of time.

Morphological Characterization Using X-ray Scattering

All X-ray experiments were conducted at beamline P03 of PETRA III (DESY, Hamburg, Germany)[8-10]. During the experiments, a fixed X-ray wavelength of 1.048 Å (*E* = 11.83 keV) and a fixed sample-detector-distance (SDD) of 21 cm was used. The beam was focused on the sample position with a size of 25 × 25 μm^2. The detector was a LAMBDA 9M unit (X-spectrum, Hamburg, Germany) with a pixel size of 55 µm.

To prevent beam damage on the samples (due to the extremely high photon flux at a 3rd generation synchrotron facility like PETRA III), they were scanned at 100 individual positions over a region of 2 × 2 mm^2 with an illumination of 1 s each. For the subsequent data processing, the images which showed the biggest similarity to each other, were summed up (Figure S18). To further analyze the scattering patterns, the 2D detector images were reduced to 1D scattering profiles (Figure S19-21). The data reduction procedure followed the well-known numerical recipes for azimuthal integration and normalization from literature.[11] The resulting scattering profiles show the radially averaged intensities over the so-called scattering vector q ($q = (4\pi/\lambda)\sin\theta$),

where λ is the X-ray wavelength and θ is the scattering angle, measured from the point where the primary beam hits the detector. After data reduction, the scattering background arising from air scattering was subtracted from each scattering profile. For modelling, the scattering profiles were normalized and a simple combination of Gaussian functions and a constant background was used. From their respective peak positions, the so-called *D*-spacing ($D = 2\pi/q$) was calculated, which gives indications about the real-space distances and sizes associated with the visible features in the scattering profiles.

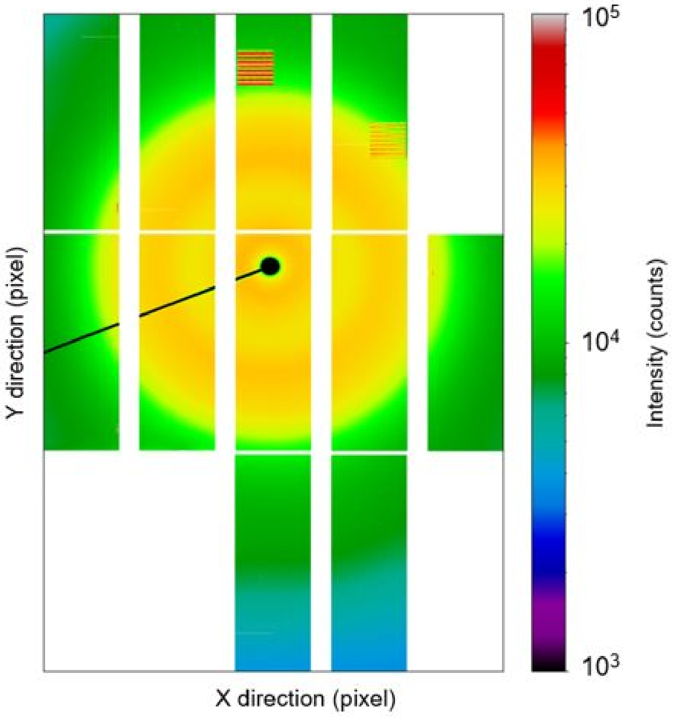

Figure S18. Summed WAXS pattern for one lignin sample. The white areas mark detector gaps or missing modules. The beam stop is visible as a black area shielding the detector from the primary X-ray beam during the experiments. For data processing, the beamstop, detector gaps, and faulty pixel areas were masked.

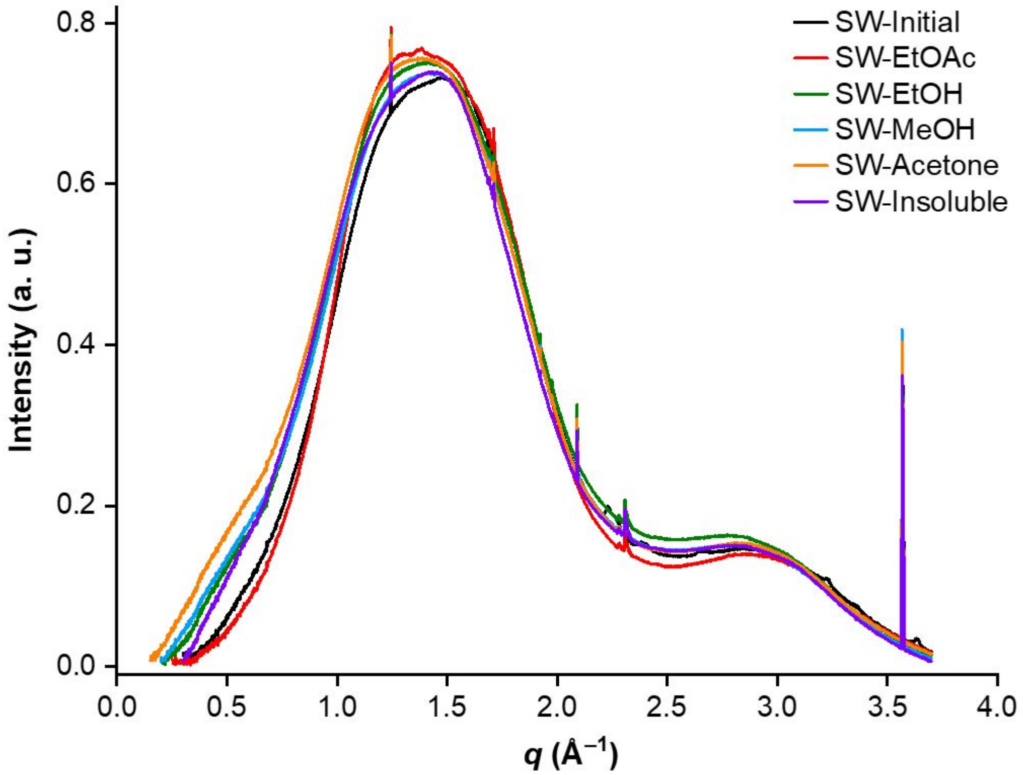

Figure S19. Wide-angle X-rays scattering signal of SW-Lignin samples.

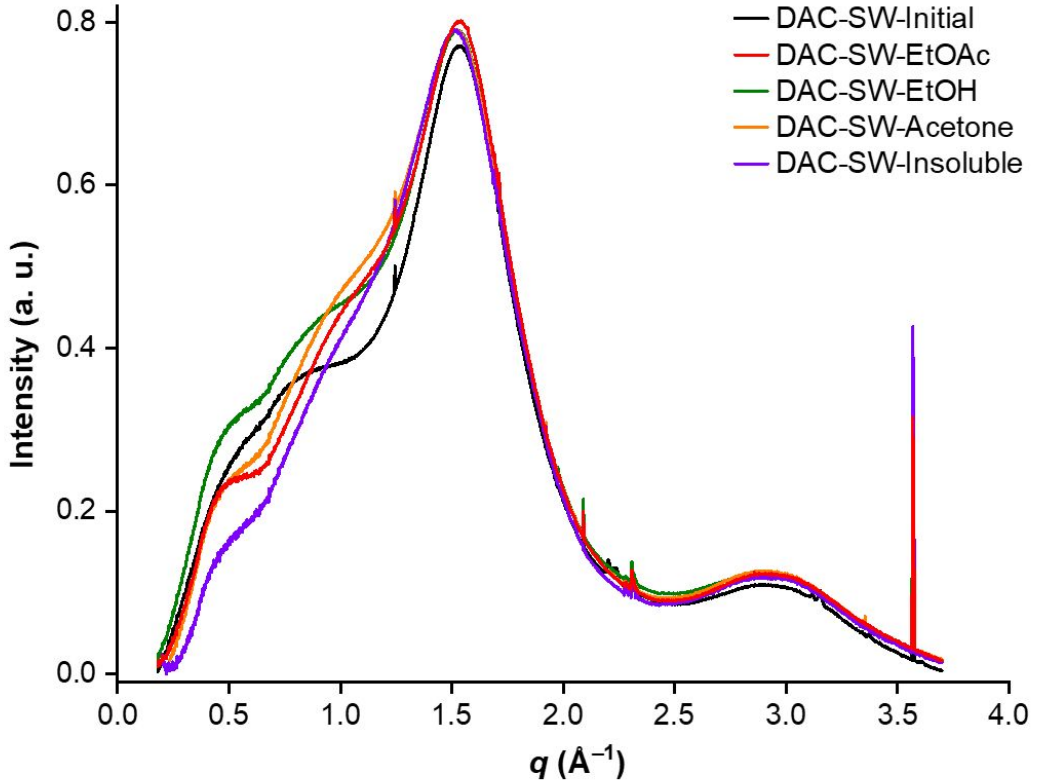

Figure S20. Wide-angle X-rays scattering signal of DAC-SW-Lignin samples.

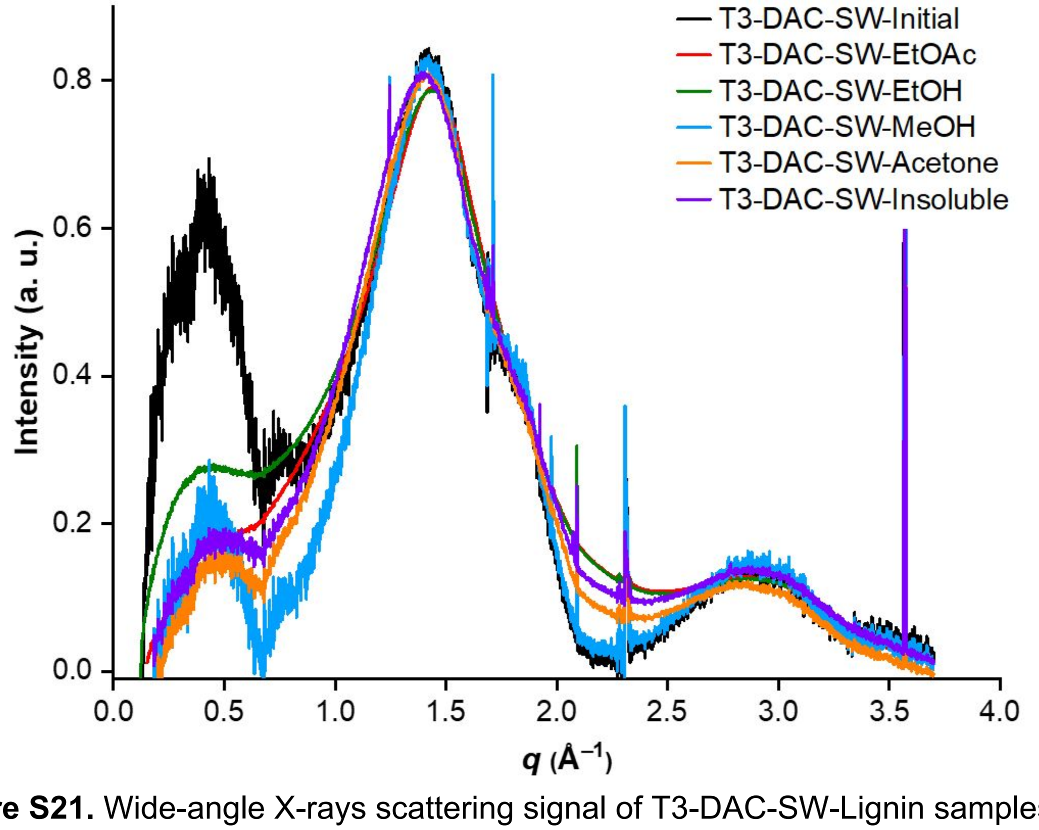

Figure S21. Wide-angle X-rays scattering signal of T3-DAC-SW-Lignin samples.

Scheme S3. Schematic description of proposed changes in scattering distances, for lignin fractions after allylation. This representation is simplified to a one aromatic ring were L represents all possible lignin structers.

π-π stacking interactions

***D_2* (T-shaped)**
- Distance:
increases by 0.53–1.33 Å
- Amount:
Increases by 4–12%

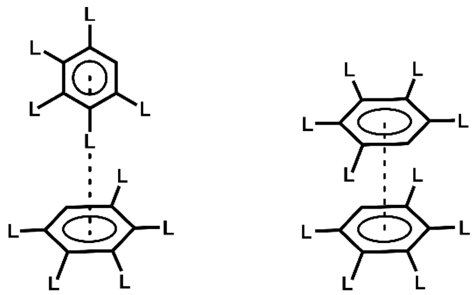

***D_3* (sandwiched)**
- Distance:
relatively constant
- Amount:
decreases by 8–16%

Table S8. Signal's maximum values of the repeating features within SW-Lignin and DAC-SW-Lignin samples determined by WAXS.

Sample	$q_{1\ max}$ (Å^{-1})	$q_{2\ max}$ (Å^{-1})	$q_{3\ max}$ (Å^{-1})	$q_{II\ order\ max\ 3}$ (Å^{-1})
SW-Initial	0.60 ± 0.01	1.07 ± 0.01	1.54 ± 0.01	2.75 ± 0.01
SW-EtOAc	0.80 ± 0.04	1.14 ± 0.01	1.52 ± 0.01	2.80 ± 0.01
SW-EtOH	0.59 ± 0.01	1.06 ± 0.01	1.50 ± 0.01	2.74 ± 0.01
SW-MeOH	0.61 ± 0.01	1.06 ± 0.01	1.48 ± 0.01	2.76 ± 0.01
SW-Acetone	0.57 ± 0.01	1.05 ± 0.01	1.47 ± 0.01	2.78 ± 0.01
SW-Insoluble	0.60 ± 0.01	0.99 ± 0.01	1.44 ± 0.01	2.75 ± 0.01
DAC-SW-Initial	0.50 ± 0.01	0.89 ± 0.01	1.53 ± 0.01	2.51 ± 0.02
DAC-SW-EtOAc	0.50 ± 0.01	0.92 ± 0.01	1.52 ± 0.01	2.90 ± 0.01
DAC-SW-EtOH	0.47 ± 0.01	0.89 ± 0.01	1.52 ± 0.01	2.62 ± 0.01
DAC-SW-MeOH			N/A	
DAC-SW-Acetone	0.49 ± 0.01	0.90 ± 0.01	1.51 ± 0.01	2.73 ± 0.01
DAC-SW-Insoluble	0.51 ± 0.01	0.91 ± 0.01	1.50 ± 0.01	2.75 ± 0.01

Table S9. Signal's maximum values of the repeating features within T3-DAC-SW-Lignin samples determined by WAXS.

Sample	$q_{1\ max}$ (Å^{-1})	$q_{2\ max}$ (Å^{-1})	$q_{3\ max}$ (Å^{-1})	$q_{4\ max}$ (Å^{-1})	$q_{II\ order\ max\ 3}$ (Å^{-1})	$q_{II\ order\ max\ 4}$ (Å^{-1})
T3-DAC-SW-Initial	0.39 ± 0.02	0.96 ± 0.41	1.44 ± 0.07	1.86 ± 0.02	2.80 ± 0.03	3.57 ± 0.06
T3-DAC-SW-EtOAc	0.43 ± 0.01	0.93 ± 0.03	1.42 ± 0.01	1.78 ± 0.25	2.80 ± 0.02	3.40 ± 0.04
T3-DAC-SW-EtOH	0.36 ± 0.01	0.87 ± 0.06	1.42 ± 0.02	1.81 ± 0.26	2.80 ± 0.02	3.40 ± 0.04
T3-DAC-SW-MeOH	0.43 ± 0.01	1.14 ± 0.66	1.45 ± 0.13	1.84 ± 0.03	2.80 ± 0.02	3.57 ± 0.04
T3-DAC-SW-Acetone	0.45 ± 0.01	1.01 ± 0.16	1.39 ± 0.03	1.74 ± 0.12	2.80 ± 0.01	3.56 ± 0.01
T3-DAC-SW-Insoluble	0.45 ± 0.03	0.93 ± 0.11	1.35 ± 0.03	1.71 ± 0.21	2.80 ± 0.02	3.53 ± 0.05

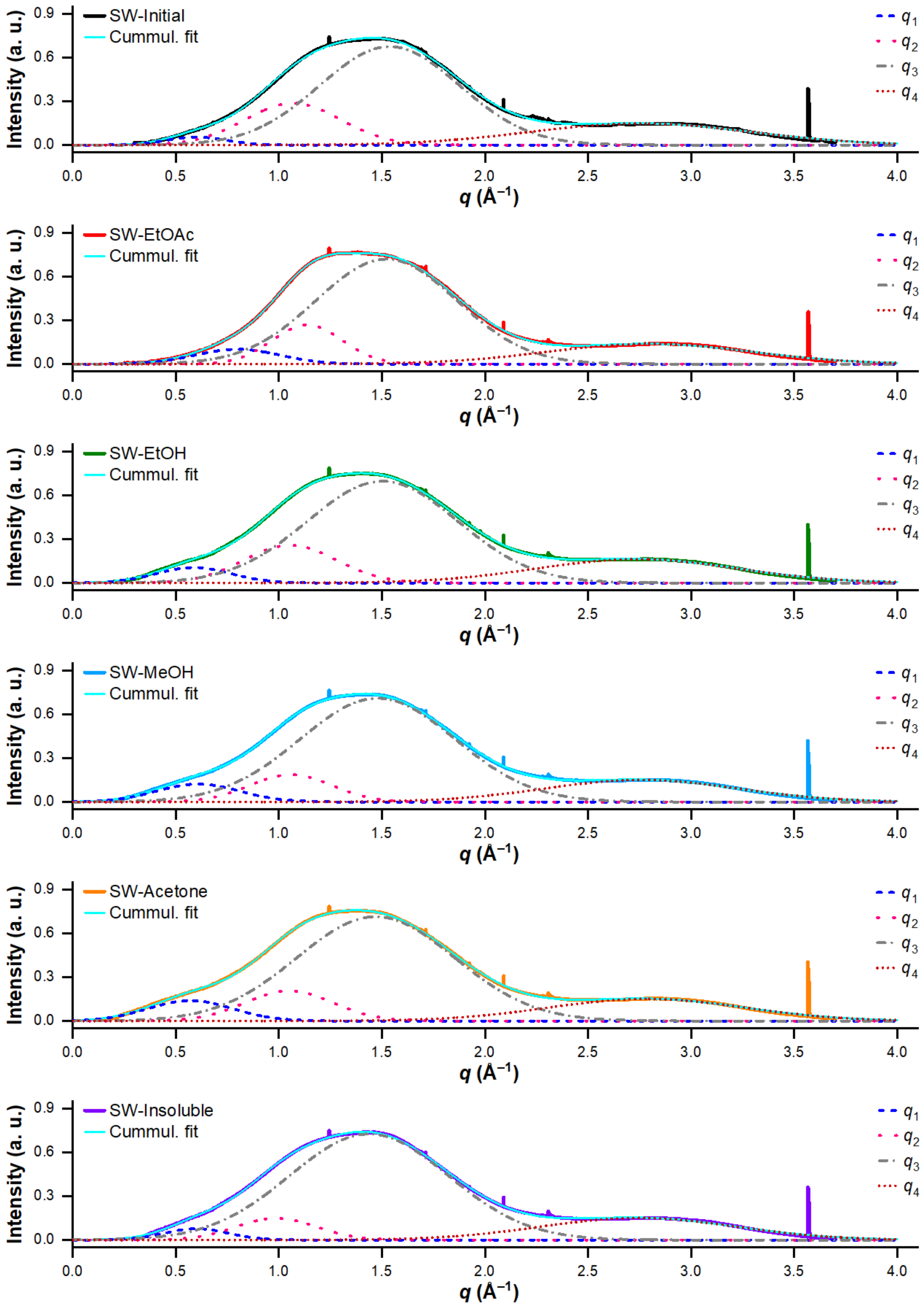

Figure S22. Fitted WAXS intensity profiles of SW-Lignin samples, using four Gaussian functions.

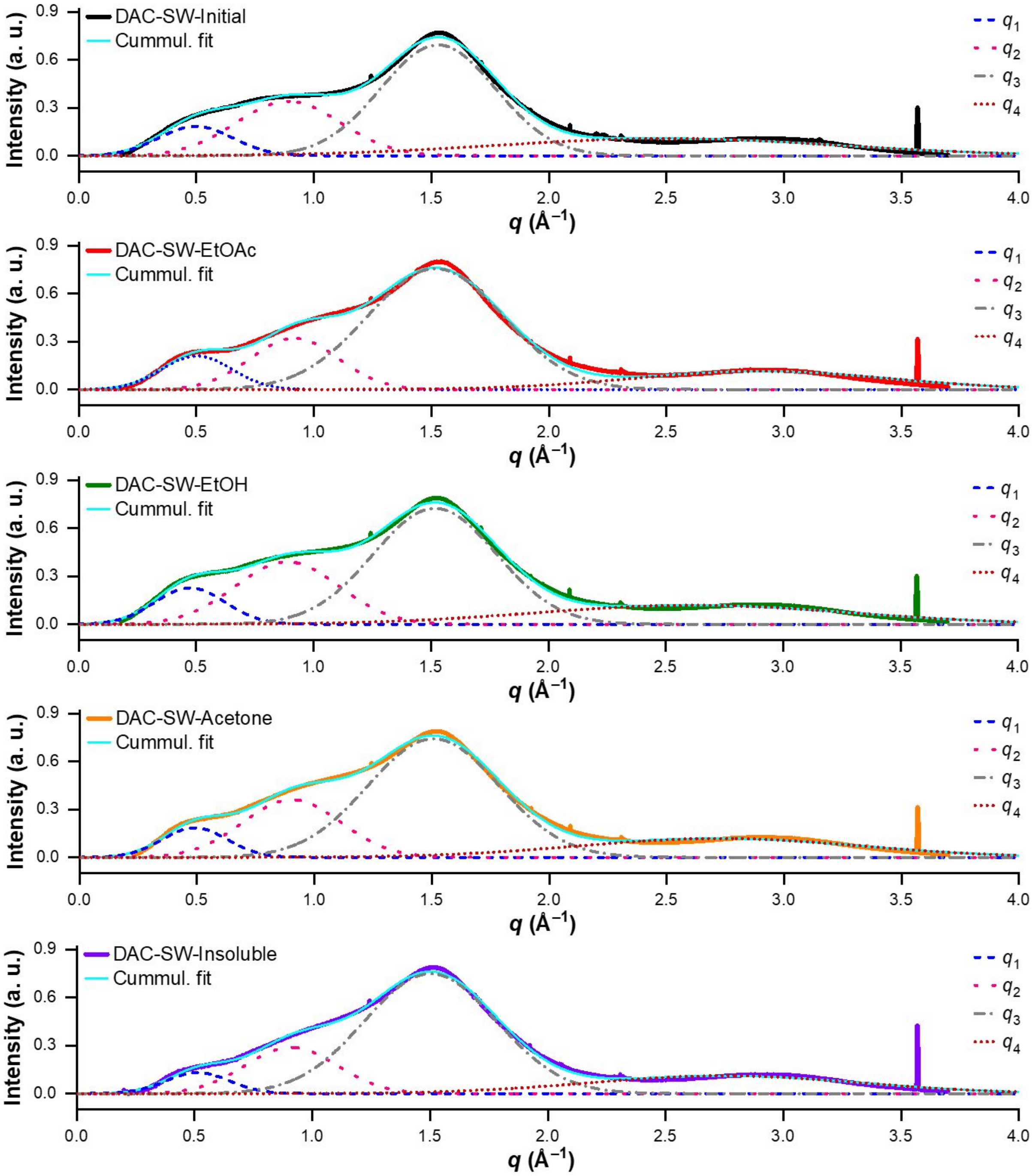

Figure S23. Fitted WAXS intensity profiles of DAC-SW-Lignin samples, using four Gaussian functions.

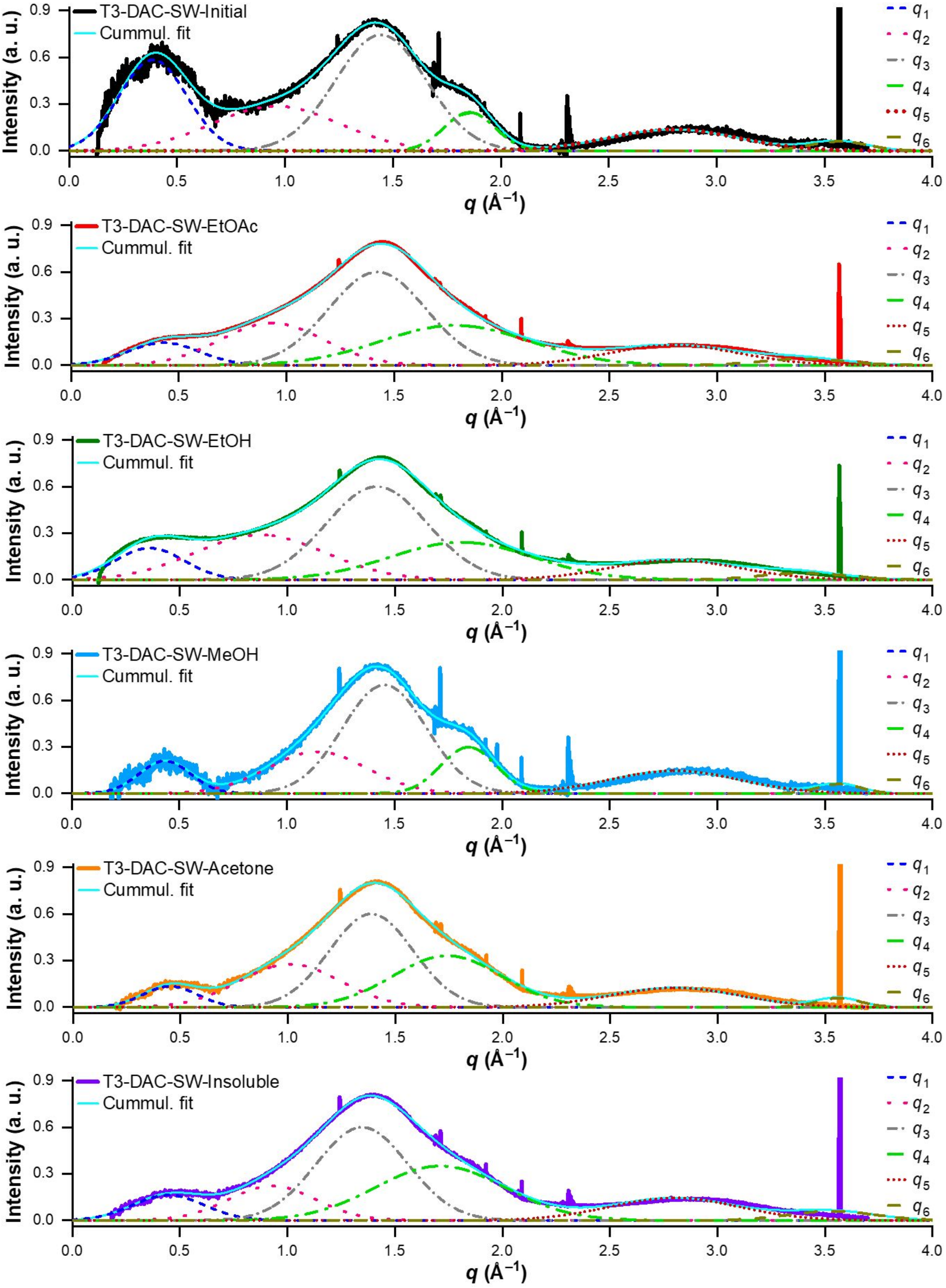

Figure S24. Fitted WAXS intensity profiles of T3-DAC-SW-Lignin samples, using four Gaussian functions.

Table S10. Contents (%) of the repeating features within SW-Lignin and DAC-SW-Lignin samples determined by WAXS. Contents were calculated by dividing the area of each signal by the sum of area of all the individual signals (q_1, q_2, and q_3) and multiplied by 100.

Sample	q_1 (%)	q_2 (%)	q_3 (%)
SW-Initial	3	22	75
SW-EtOAc	7	15	79
SW-EtOH	6	17	77
SW-MeOH	8	11	81
SW-Acetone	8	13	79
SW-Insoluble	4	9	87
DAC-SW-Initial	11	28	62
DAC-SW-EtOAc	10	19	71
DAC-SW-EtOH	11	28	61
DAC-SW-MeOH		N/A	
DAC-SW-Acetone	8	25	67
DAC-SW-Insoluble	6	20	74

Table S11. Contents (%) of the repeating features within T3-DAC-SW-Lignin samples determined by WAXS. Content (%) was calculated by dividing the area of each signal by the sum of area of all the individual signals (q_1, q_2, q_3, and q_4) and multiplied by 100.

Sample	q_1 (%)	q_2 (%)	q_3 (%)	q_4 (%)
T3-DAC-SW-Initial	25	23	43	8
T3-DAC-SW-EtOAc	8	21	43	28
T3-DAC-SW-EtOH	10	26	41	24
T3-DAC-SW-MeOH	10	24	52	15
T3-DAC-SW-Acetone	6	23	41	30
T3-DAC-SW-Insoluble	9	15	42	35

Mechanical Properties of the Thermosets

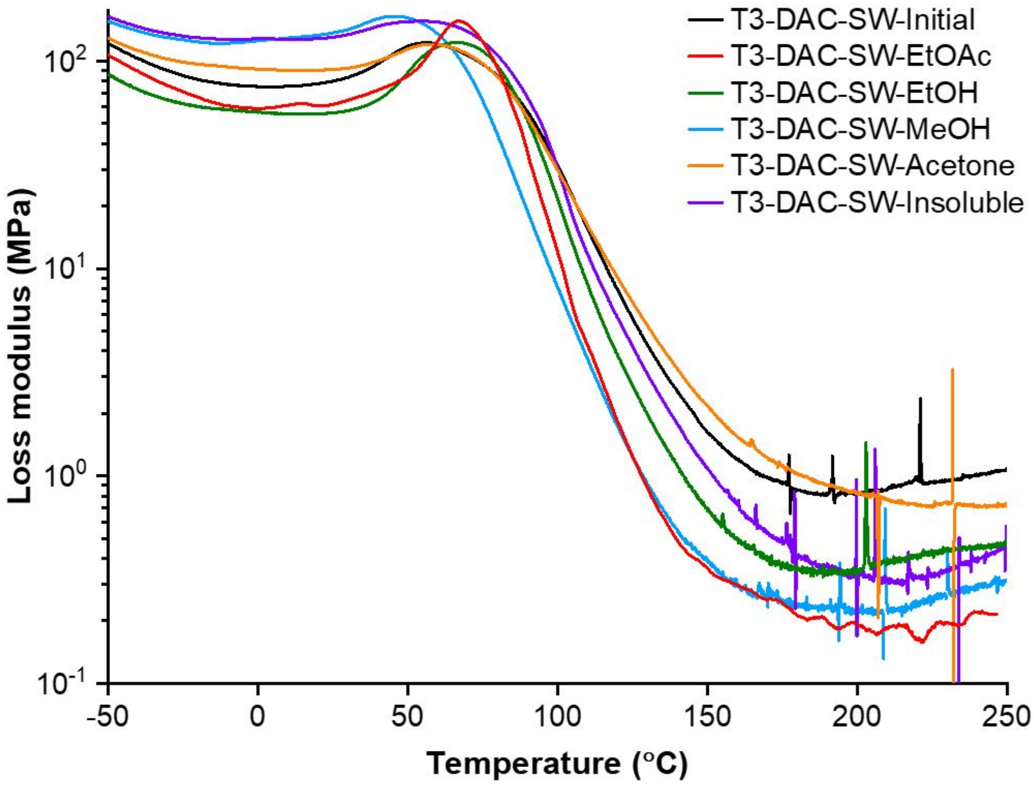

Figure S25. Loss modulus (E'') of representative DMA curves of different lignin-based thermosets.

Table S12. Strain at break (ε_b), stress at break (σ_b), and Young's modulus (E) extracted from tensile stress-strain curves.

Sample	ε_b (%)	σ_b (MPa)	E (GPa)
T3-DAC-SW-Initial	5.4 ± 2.9	29.9 ± 13.2	1.38 ± 0.12
T3-DAC-SW-EtOAc	2.5 ± 1.5	31.6 ± 15.1	1.95 ± 0.16
T3-DAC-SW-EtOH	5.8 ± 2.8	49.0 ± 18.7	2.32 ± 0.16
T3-DAC-SW-MeOH	3.2 ± 1.2	30.2 ± 9.9	1.65 ± 0.14
T3-DAC-SW-Acetone	3.7 ± 0.6	33.4 ± 4.9	1.82 ± 0.27
T3-DAC-SW-Insoluble	1.6 ± 0.2	18.9 ± 9.6	2.05 ± 0.15

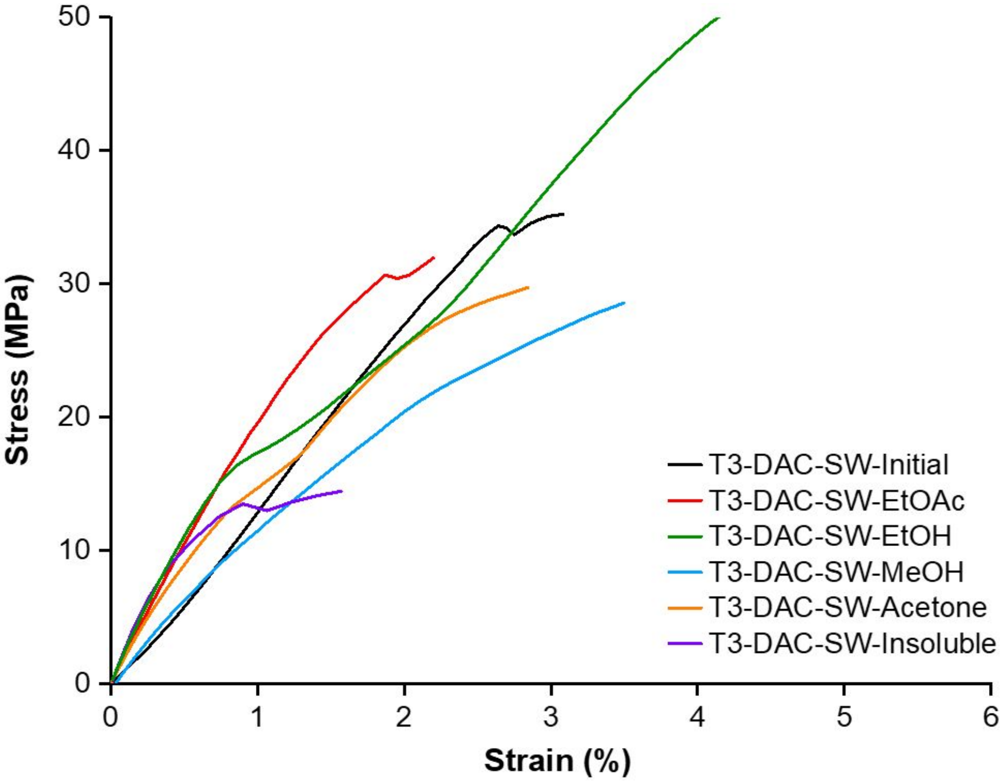

Figure S26. Representative tensile stress-strain curves for the different lignin-based thermosets.

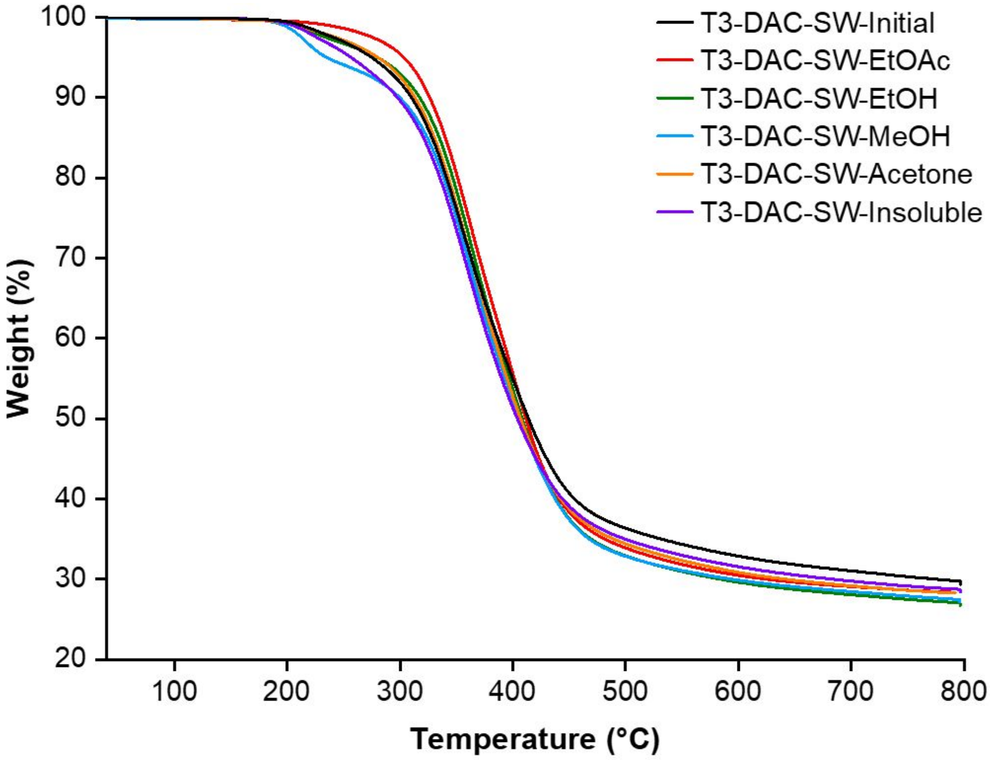

Figure S27. Representative TGA curves of different lignin-based thermosets.

References

1. Duval, A; Vilaplana, F; Crestini, C; Lawoko, M, Solvent screening for the fractionation of industrial kraft lignin. *Holzforschung* **2016,** *70* (1), 11-20 DOI: https://10.1515/hf-2014-0346
2. Ribca, I; Jawerth, M E; Brett, C J; Lawoko, M; Schwartzkopf, M; Chumakov, A; Roth, S V; Johansson, M, Exploring the Effects of Different Cross-Linkers on Lignin-Based Thermoset Properties and Morphologies. *ACS Sustainable Chemistry & Engineering* **2021,** *9* (4), 1692-1702 DOI: https://10.1021/acssuschemeng.0c07580
3. Over, L C; Meier, M A R, Sustainable allylation of organosolv lignin with diallyl carbonate and detailed structural characterization of modified lignin. *Green Chemistry* **2016,** *18* (1), 197-207 DOI: https://10.1039/c5gc01882j
4. Jawerth, M; Johansson, M; Lundmark, S; Gioia, C; Lawoko, M, Renewable Thiol–Ene Thermosets Based on Refined and Selectively Allylated Industrial Lignin. *ACS Sustainable Chemistry & Engineering* **2017,** *5* (11), 10918-10925 DOI: https://10.1021/acssuschemeng.7b02822
5. Meng, X; Crestini, C; Ben, H; Hao, N; Pu, Y; Ragauskas, A J; Argyropoulos, D S, Determination of hydroxyl groups in biorefinery resources via quantitative (31)P NMR spectroscopy. *Nature Protocols* **2019,** *14* (9), 2627-2647 DOI: https://10.1038/s41596-019-0191-1
6. Huang, Y; Wang, L; Chao, Y; Nawawi, D S; Akiyama, T; Yokoyama, T; Matsumoto, Y, Analysis of Lignin Aromatic Structure in Wood Based on the IR Spectrum. *Journal of Wood Chemistry and Technology* **2012,** *32* (4), 294-303 DOI: https://10.1080/02773813.2012.666316
7. Over, L C. Sustainable Derivatization of Lignin and Subsequent Synthesis of Cross-Linked Polymers. Karlsruhe Institute of Technology (KIT), 2017.
8. Roth, S V; Herzog, G; Korstgens, V; Buffet, A; Schwartzkopf, M; Perlich, J; Abul Kashem, M M; Dohrmann, R; Gehrke, R; Rothkirch, A; Stassig, K; Wurth, W; Benecke, G; Li, C; Fratzl, P; Rawolle, M; Muller-Buschbaum, P, In situ observation of cluster formation during nanoparticle solution casting on a colloidal film. *Journal of Physics: Condensed Matter* **2011,** *23* (25), 254208 DOI: https://10.1088/0953-8984/23/25/254208
9. Buffet, A; Rothkirch, A; Dohrmann, R; Korstgens, V; Abul Kashem, M M; Perlich, J; Herzog, G; Schwartzkopf, M; Gehrke, R; Muller-Buschbaum, P; Roth, S V, P03, the microfocus and nanofocus X-ray scattering (MiNaXS) beamline of the PETRA III storage ring: the microfocus endstation. *Journal of Synchrotron Radiation* **2012,** *19* (Pt 4), 647-53 DOI: https://10.1107/S0909049512016895
10. Krywka, C; Neubauer, H; Priebe, M; Salditt, T; Keckes, J; Buffet, A; Roth, S V; Doehrmann, R; Mueller, M, A two-dimensional waveguide beam for X-ray nanodiffraction. *Journal of Applied Crystallography* **2011,** *45* (1), 85-92 DOI: https://10.1107/s0021889811049132
11. Pauw, B R; Smith, A J; Snow, T; Terrill, N J; Thunemann, A F, The modular small-angle X-ray scattering data correction sequence. *Journal of Applied Crystallography* **2017,** *50* (Pt 6), 1800-1811 DOI: https://10.1107/S1600576717015096
